# Effects of cancer-associated point mutations on the structure, function, and stability of isocitrate dehydrogenase 2

**DOI:** 10.1038/s41598-022-23659-y

**Published:** 2022-11-05

**Authors:** Xiang Chen, Peipei Yang, Yue Qiao, Fei Ye, Zhipeng Wang, Mengting Xu, Xiaowang Han, Li Song, Yuehong Wu, Wen-Bin Ou

**Affiliations:** Zhejiang Provincial Key Laboratory of Silkworm Bioreactor and Biomedicine, College of Life Sciences and Medicine, Zhejiang Sci-Tech University, Hangzhou, China

**Keywords:** Biochemistry, Cancer, Structural biology

## Abstract

Mutations in isocitrate dehydrogenase (IDH) are frequently found in low-grade gliomas, secondary glioblastoma, chondrosarcoma, acute myeloid leukemias, and intrahepatic cholangiocarcinoma. However, the molecular mechanisms of how IDH2 mutations induce carcinogenesis remain unclear. Using overlapping PCR, transfection, immunoblotting, immunoprecipitation, measurements of enzyme activity, glucose, lactic acid, ATP, and reactive oxygen species (ROS), cell viability, protein degradation assays post-inhibition of the 26S proteasome (bortezomib) or HSP90 (17-AAG), and a homology model, we demonstrated that the properties of ten cancer-associated IDH2 variants (R140G/Q/W and R172S/K/M/W/G/C/P) arising from point mutations are closely related to their structure and stability. Compared with wild-type IDH2, the R172 and R140 point mutations resulted in a decrease in IDH2 activity, ROS, and lactate levels and an increase in glucose and ATP levels under normal and hypoxic conditions, indicating that mutant IDH2 increases cell dependency on mitochondrial oxidative phosphorylation, and reduces glycolysis under hypoxia. Overexpression of most of IDH2 point mutants showed anti-proliferative effects in the 293T and BV2 cell lines by inhibition of PI3K/AKT signaling and cyclin D1 expression and/or induced the expression of TNF-α and IL-6. Furthermore, bortezomib treatment resulted in dramatic degradation of IDH2 mutants, including R140G, R140Q, R140W, R172S and R172K, whereas it had little impact on the expression of WT and other mutants (R172M, R172W, R172G, R172C and R172P). In addition, targeting HSP90 minimally affected the expression of mutated IDH2 due to a lack of interaction between HSP90 and IDH2. The homology model further revealed that changes in conformation and IDH2 protein stability appeared to be associated with these point mutations. Taken together, our findings provide information important for understanding the molecular mechanisms of IDH2 mutations in tumors.

## Introduction

Isocitrate dehydrogenase (IDH) is an important enzyme for cellular metabolism that is part of the Krebs cycle. *IDH* codes for three isoforms (IDH1, IDH2 and IDH3). IDH2 catalyzes the oxidative decarboxylation of isocitrate to form α-ketoglutarate (α-KG) and NADPH using NADP^+^ as a cofactor in mitochondria^1^. To date, the IDH crystal structures of wild-type (WT) or mutants from prokaryotes and humans complexed with various ligands/inhibitors^2–5^ or from porcine heart mitochondrial IDH (PcIDH, PDB ID: 1LWD) with a 97% homologous sequence to human IDH2^6^ have been resolved, such as IDH1-R132H^2^, IDH2-R140Q^3^, and IDH2-R172K^4^.

However, the structure of *Homo sapiens* IDH2 and other point mutants associated with cancers remains unclear. Numerous studies have shown that IDH1/IDH2 mutations frequently occur in low-grade gliomas^7^, secondary glioblastoma^8^, chondrosarcoma^9^, acute myeloid leukemia (AML)^10^, and intrahepatic cholangiocarcinoma^11^. Point mutations are mainly found in the arginine residues within the isocitrate binding sites of *IDH* active sites*,* including IDH1/R132, IDH2/R140, and IDH2/R172.

Although their mechanisms of tumorigenesis have not been fully demonstrated, IDH mutations have been found to convert α-KG to the oncometabolite 2-hydroxyglutarate (2-HG), which dysregulates a set of α-KG-dependent dioxygenases^12^. Specifically, due to the similar structure between 2-HG and α-KG, 2-HG competitively blocks the binding of α-KG to histone demethylases, such as lysine-specific demethylase (KDM), and then it increases histone methylation^13^. 2-HG also inhibits the activity of ten-eleven translocation hydroxylases, which are important for global DNA methylation^14^. In addition, the accumulated 2-HG results in inhibition of prolyl-hydroxylase (PHD) activity and induction of pyruvate dehydrogenase kinase (PDK), which hydroxylates hypoxia-inducible factor 1α subunit (HIF-1α) at specific proline residues and increases HIF-1α expression. Furthermore, the high levels of 2-HG because of IDH mutations stabilize HIF1α by decreasing levels of the HIF-1α antagonist endostatin, which results in upregulation of HIF1α-dependent genes, including vascular endothelial growth factor (VEGF)^15,16^.

Recently studies have found that the point mutation frequency of IDH2 is relative lower than that of IDH1, for example, in glioma (1.6–26.8%), intrahepatic cholangiocarcinoma (5.7–23.1%), and chondrosarcoma (10.7–19.4%), IDH2 R172 mutants include R172K (39%), R172S (29%), R172W (12%), R172G (10%), R172M (5%), and R172T (4%)^11^. R140Q (17.9%) and R172K (20.2%) are more common point mutations in AML^17^. R140 and R172 mutant proteins possess neomorphic enzymatic activity resulting in 2-HG accumulation, indicating that both mutations are associated with cancer progression.

Mutated IDH2 has been found to be an important therapeutic target in above-mentioned cancers. However, it is still unclear for the molecular mechanism of pathogenesis, specially, the conformational changes and stability of the mutated IDH2 protein associated with function. In the present study, the effects of ten very common IDH2 point mutations (R140G/Q/W and R172S/K/M/W/G/C/P) on the function, structure, and stability of protein were evaluated by the measurement of enzyme activity, glucose, lactate, ATP, and ROS levels, degradation analysis post-treatment with 26S proteasome inhibitor (bortezomib) or HSP90 inhibitor (17-AAG), and bioinformatics homology modeling. The current data demonstrated that IDH2 point mutations reduced their enzymatic activity and protein stability due to conformation changes. These data provide important information for understanding the molecular mechanisms of IDH2 mutations in tumorigenesis.

## Materials and methods

### Antibodies and reagents

Phospho-AKT, polyclonal antibodies against AKT and TNF-α, and the monoclonal antibody against S6 were from Cell Signaling Technology (Beverly, MA). Monoclonal mouse antibodies against cyclin D1, IL6, and HSP90 (Santa Cruz Biotechnology, CA), GAPDH (Abcam Biotechnology, Cambridge, MA), β-actin (Sigma-Aldrich, St. Louis, MO), and His (Quanshijin Biotechnology, Beijing, China) were also used.

Protein A-sepharose beads and protein G-sepharose beads were purchased from Santa Cruz Biotechnology. Bortezomib was obtained from Selleck (Shanghai, China), and 17-AAG and CoCl_2_·6H_2_O were obtained from Sigma-Aldrich. Lipofectamine and PLUS reagent were purchased from Invitrogen Life Technologies. The wild-type prokaryotic *IDH2* expression vector (EX-C0462-B01) was from FulenGen Co., Ltd. (Guangzhou, China). The empty eukaryotic vector pCMV6-AC-Myc-His was purchased from Origene Technologies (Rockville, MD). The restriction endonucleases *Hind*III and *Mlu*I were obtained from New England Biolabs (MA). Premix PrimeSTAR HS, QuickCut enzyme, T4 DNA ligase, DNA Marker, and DNA Loading Buffer were purchased from Takara (Kusatsu, Japan). Primer synthesis (Table S1) and sequencing were completed by Sangon Biotech (Shanghai, China).

### Cell culture

HEK293T cell line was a kind gift from Dr. Jonathan A. Fletcher in the Laboratory of Oncology at Brigham and Women’s Hospital and Harvard Medical School. The mouse microglial cell line BV2 was purchased from the cell library of the Chinese Academy of Sciences in Shanghai. The HEK293T cell line was cultured in RPMI 1640 medium with 10% fetal bovine serum (FBS) supplemented with 1% (v/v) penicillin/streptomycin and 1% (v/v) L-glutamine, and the mouse microglial BV2 cell line was maintained in DMEM/F-12 (Gibco) with 10% FBS supplemented with 1% (v/v) penicillin/streptomycin and 1% (v/v) L-glutamine at 37 °C in a 5% CO_2_ humidified atmosphere. The HEK293T cell line and BV2 cell line were regularly screened for mycoplasma contamination using a Mycoplasma Stain Assay Kit (Beyotime Biotechnology, Shanghai).

### Generation of *IDH2* WT and point mutant vectors

**Figure 1:**
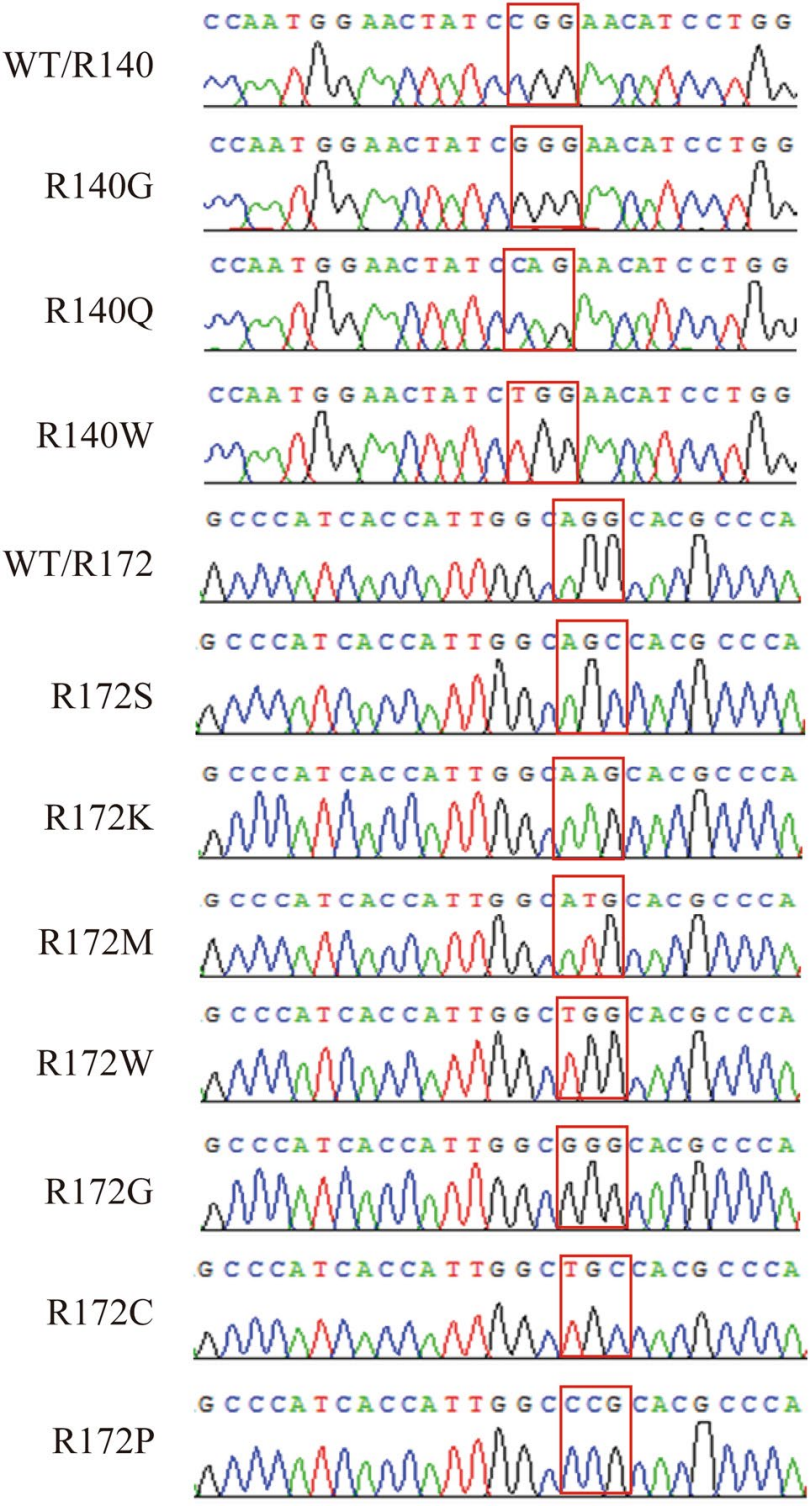
Analysis of the recombinant *IDH2* wild-type and mutant sequences. The sequences of wild-type, R140-mutated and R172-mutated *IDH2* were corrected by BLASTing against GenBank.

The *IDH2* point mutants and PCR primers are listed in Table S1. PCR was performed in a reaction volume of 25 μL with Kapa HiFi HotStart 2 × MIX. The reactions contained 200 ng DNA, 12.5 pmol of each primer and 12.5 μl Kapa HiFi HotStart 2 × MIX. After 3 min at 95 °C, each of the 30 cycles consisted of denaturation for 20 s at 95 °C and annealing at 60 °C for 20 s. The WT *IDH2* target fragment was obtained by PCR, and *IDH2* was cloned into the *Hind*III and *Mlu*I restriction sites of the mammalian vector (pCMV6-AC-Myc-His). *IDH2* point mutants, including R140G, R140Q, R140W, R172S, R172K, R172M, R172W, R172G, R172C, and R172P, were constructed by nested PCR based on the WT. All constructs were verified to contain the correct point mutation by restriction analysis and sequencing (Fig. 1).

### Transfection

Transfection of *IDH2* WT and mutant constructs into the HEK293T or BV2 cell lines was carried out according to the manufacturer’s instructions by using Lipofectamine and PLUS reagent. Briefly, 1 µg DNA was incubated with PLUS reagent in 100 µl of serum-free medium (SFM) for 15 min at room temperature (RT), then the Lipofectamine was diluted with 100 µl of SFM and added to DNA and PLUS complexes, and incubated for another 15 min at RT. Finally, the DNA-PLUS-Lipofectamine complexes were added to 60% confluent cultures of 293T or BV2 cells in 800 µL of SFM in six-well plates. The DNA-PLUS-Lipofectamine complexes in SFM were completely replaced with serum-containing regular media after 3 h of incubation. Cells were lysed for immunoblotting or functional analysis, including measurement of IDH2 enzymatic activity, glucose, and lactic acid levels at 48 or 72 h post-transfection.

### Protein lysate preparations and immunoblotting

Immunoblotting was performed 48 h post-transfection or after treatment with 17-AAG or bortezomib for 72 h. Whole cell lysates were prepared using lysis buffer (50 mM Tris–HCl pH 8.0, 100 mM sodium fluoride, 30 mM sodium pyrophosphate, 2 mM sodium molybdate, 5 mM EDTA, 2 mM sodium orthovanadate, 1% NP-40, 10 μg/mL aprotinin, 10 μg/mL leupeptin, and 1 mM phenylmethylsulfonyl fluoride). Lysates were cleared by centrifugation at 15,000 rpm for 30 min at 4 °C, and the supernatant protein concentrations were determined using a Bio–Rad protein assay (Bio–Rad Laboratories Hercules, CA). Electrophoresis and western blotting were performed as described previously^18^. The hybridization signals were detected by chemiluminescence (Immobilon™ Western, Millipore Corporation, MA) and captured using a GE ImageQuant LAS4000 chemiluminescence imaging system.

### Immunoprecipitation

First, 1 mg of protein lysate was precleared for 30 min at 4 °C using 20 μL of protein G/A beads. Three micrograms of primary mouse antibodies against His and primary rabbit antibodies against HSP90 were rocked with the lysates for 2–3 h at 4 °C. Next, 20 μL of Sepharose-protein G/A beads was added and incubated overnight at 4 °C and then centrifuged at 10,000 rpm for 2 min, after which the Sepharose beads were washed 3 times with 750 μL of IP buffer for 10 min and once with 750 μL of 10 mM Tris–HCl buffer (pH 7.6). Finally, loading buffer (20 μL) was added to the beads and they were boiled for 5 min at 95 °C. The interaction of IDH2 and HSP90 was subsequently evaluated by immunoblotting with specific antibodies.

### Measurement of IDH2 enzymatic activity

293T cells were lysed 48 h post-transfection. The lysates were sonicated and centrifuged at 12,000 rpm at 4 °C. Supernatants were then collected and normalized to the total protein concentration. The oxidative activity of IDH2 was determined by monitoring the increase in the absorbance of NAPDH at 340 nm over a period of 60 min on a Thermo Scientific Microplate Reader. The reaction mixtures consisted of 5.2 μg of lysate protein, 100 mM Tris–Cl buffer (pH 7.5), 1.3 mM MnCl_2_, 0.33 mM EDTA, 0.1 mM β-NADP^+^, and d-(+)-*threo*-isocitrate in a total volume of 200 μL. All assays were performed in triplicate.

### Analysis of glucose, lactic acid, and ATP levels

HEK293T cells were lysed 48 h post-transfection with *IDH2* WT and mutant constructs under normal and/or hypoxic conditions (200 µM CoCl_2_ treatment). Changes in intracellular lactic acid and glucose levels were determined with the Lactic Acid Assay Kit (Jiancheng Bioengineering Institute, Nanjing), the Glucose Assay Kit (Rongsheng Biological Pharmaceutical Co. Shanghai), and the ATP Detection Kit (Beyotime Biotechnology Co. Shanghai). According to the manufacturer`s instructions, the changes in the absorption value at 505 nm (glucose) or 530 nm (lactic acid) were measured by UV spectrophotometry (UV-1800PC, MAPADA) after the lysate proteins (5.2 μg/μL) were added to the working solution. ATP levels were measured by Luminometer (Lumat LB 9507, Berthold technologies) after the lysate proteins (2 μg/μL) were added to the working solution. All assays were performed in triplicate. The data were finally analyzed and exported by GraphPad Prism 9.0.0 (GraphPad Software, LLC).

### Measurement of ROS levels

According to the manufacturer`s protocol, ROS levels were measured using the Reactive Oxygen Species Assay Kit (Beyotime Biotechnology Co. Shanghai) under normal or hypoxic conditions (200 µM CoCl_2_ treatment). Namely, the oxidative conversion of cell-permeable dichlorofluorescein diacetate (DCFH-DA) to fluorescent dichlorofluorescein (DCF) indicates changes in intracellular ROS levels. Briefly, 293 T cells in 6-well culture dishes were incubated with 10 μM DCFH-DA at 37 °C for 20 min at 48 h post-transfection using *IDH2* WT and point mutant constructs, and then the DCF fluorescence distribution of the cells was detected at a 488 nm excitation wavelength and a 535 nm emission wavelength by a fluorospectrophotometer (F4000, Japan) or at FITC channel by a flow cytometry (V6B5R3, Agilent) after the cells were washed twice with PBS. All assays were performed in triplicate. The data were finally analyzed and exported by NovoExpress (Agilent Technologies, lnc).

### Cell viability analysis

293T cells were plated at 3000 cells per well in a 96-well flat-bottomed plate (Greiner, Frickenhausen, Germany) and cultured in RPMI 1640 for 24 h before transfection with *IDH2* WT and mutation constructs. Proliferation studies were carried out after 6 days using the MTT assay, and quantified using a Microplate Reader (TECAN, Austria). The data were normalized to WT. All the assays were performed in triplicate wells, and at least three independent experiments were performed.

### Bioinformatics homology model

Tertiary structure predictions were made with the published crystal structure of porcine heart mitochondrial isocitrate dehydrogenase (PcIDH, PDB ID: 1LWD)^6^ using a modeler. Structural images of IDH2 WT and mutant proteins were generated using PYMOL.

### Statistical analysis

Student’s t-tests were performed on data from cells treated with empty vector or IDH2 WT/point mutants. Statistically significant differences between WT and mutations were defined as *p < 0.05, **p < 0.01, ***p < 0.001, and ****p < 0.0001.

## Results

### Overexpression of IDH2 wild-type and mutants

**Figure 2:**
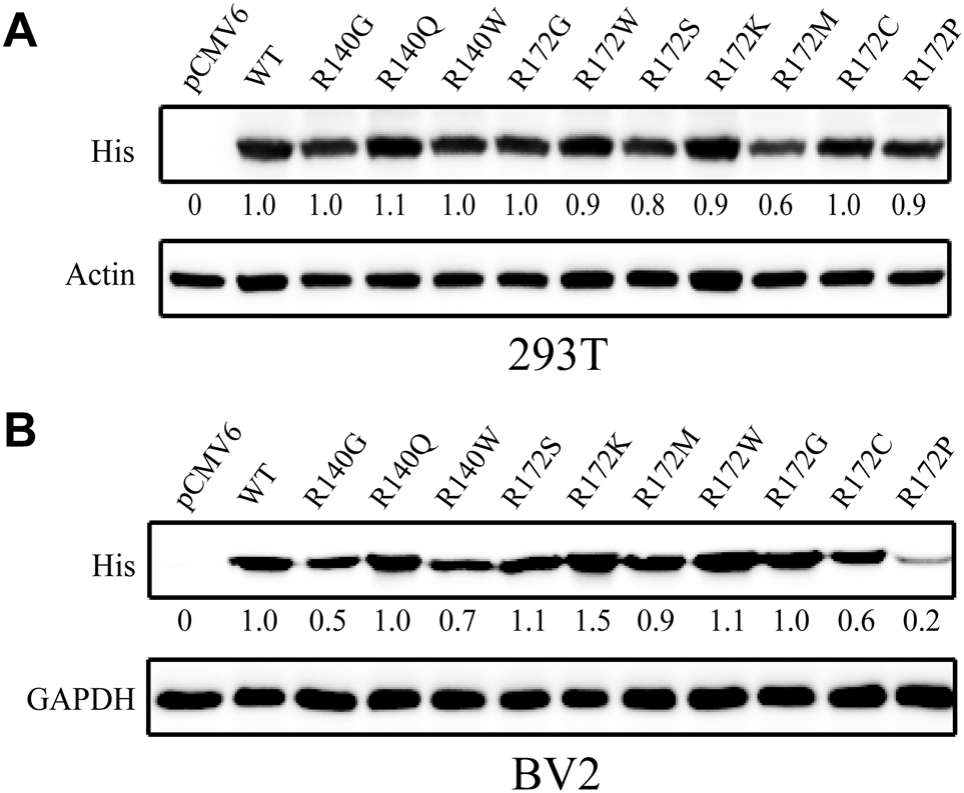
Overexpression of IDH2 WT and mutants in the 293T or BV2 cell line was assessed by western blotting using anti-His antibody at 48 h posttransfection. Actin or GAPDH staining was used as a loading control.

IDH2 mutations occur frequently in a variety of tumors, including AML, brain tumors, and glioma. Point mutations occur in the active-site arginine residues of IDH2, such as R140 and R172. To demonstrate the IDH2 function associated with the conformational changes and stability of the mutant protein, IDH2 WT and ten cancer-associated IDH2 variants (R140G/Q/W and R172S/K/M/W/G/C/P) were overexpressed in 293T and BV2 cell lines by transfection. Immunoblotting showed that the expression of IDH2 WT and R140/R172 mutants was increased compared to that of the pCMV6 empty vector. According to the gray value of the IDH2 protein bands, the overexpression level of each IDH2 mutant was comparable to that of the WT protein (Fig. 2).

### Point mutations result in IDH2 inactivation

**Figure 3:**
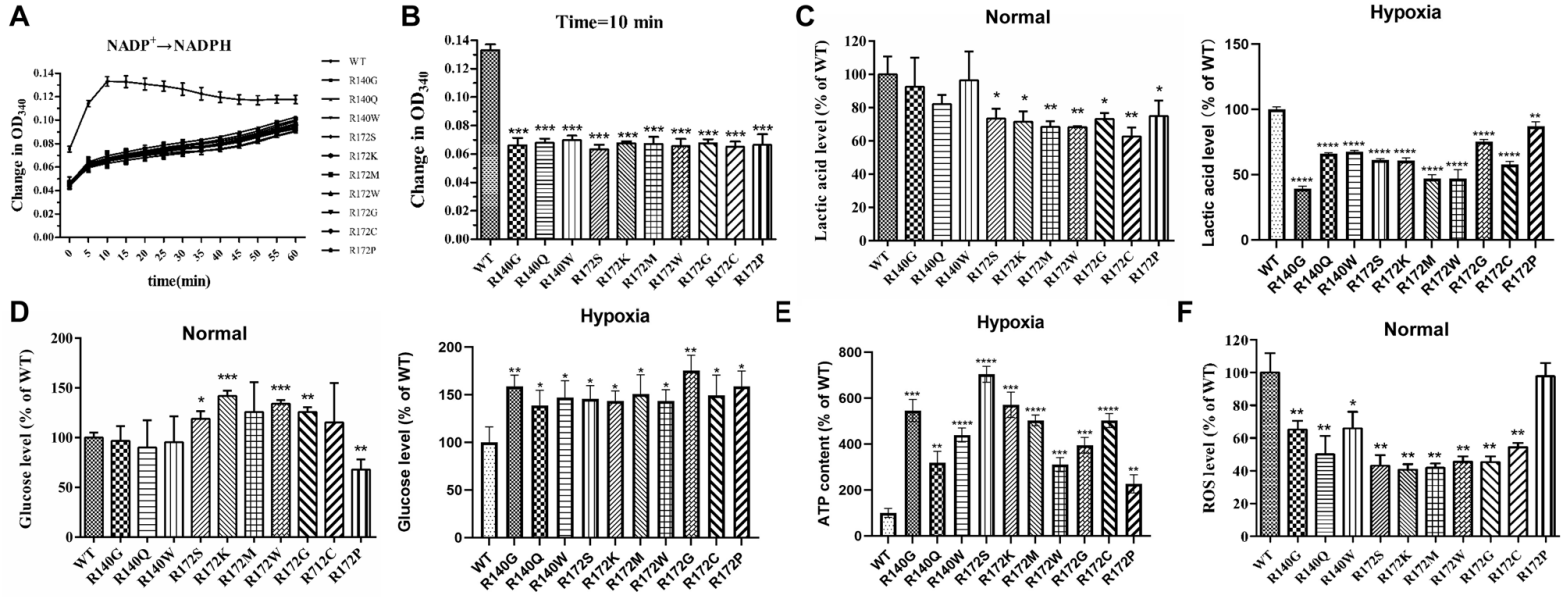
Analysis of the enzymatic activity of the mutated IDH2 protein from total 293T cell lysates at 48 h posttransfection. (**A**) The oxidative activity of IDH2 was determined by monitoring the increase in the absorbance of NAPDH at 340 nm over a period of 60 min on a Thermo Scientific Microplate Reader. (**B**) Assay of the maximum IDH2 activity in the tenth minute. Data were normalized to the IDH2 WT group and represent the mean values (± SD) from quadruplicate measurements. Statistically significant differences between WT and mutants are presented as ***p < 0.001. (**C**,**D**) Changes in intracellular lactic acid and glucose levels were determined under normal and hypoxic conditions by using lactic acid and glucose assay kits. The changes in the absorption value at 505 nm (glucose) or 530 nm (lactic acid) were measured by UV spectrophotometry. Statistically significant differences between WT and mutants are presented as *p < 0.05, **p < 0.01, ***p < 0.001, ****p < 0.0001. (**E**) ATP levels were measured in 293 T under hypoxia conditions by using the ATP Detection Kit and Luminometer at 48 h post-transfection with *IDH2* WT and mutation constructs. Data were normalized to WT and represent the mean values (± s.d.) of triplicate cultures. All the assays were performed from triplicate experiments. Statistically significant differences between WT and mutants are presented as **p < 0.01, ***p < 0.001, ****p < 0.0001. (**F**) ROS levels were measured under normal conditions by using a Reactive Oxygen Species Assay Kit and a fluorospectrophotometer. The oxidative conversion of cell permeable dichlorofluorescein diacetate (DCFH-DA) to fluorescent dichlorofluorescein (DCF) indicates changes in intracellular ROS levels. Statistically significant differences between wild-type and mutations are presented as *p < 0.05, **p < 0.01, ***p < 0.001. All assays were performed in triplicate.

To investigate the effect of the point mutations on IDH2 function, we firstly compared the differences in enzymatic activity between IDH2 WT and mutants (Fig. 3A). As described previously, the enzymatic activity of IDH2 from total 293T cell lysates 48 h post-transfection with WT or mutated *IDH2* was determined by monitoring the increase in the absorbance of NAPDH at 340 nm. The enzymatic activity of WT IDH2 reached its maximum value during the tenth minute (Fig. 3A). Compared to that of the WT protein, the point mutations in IDH2 resulted in an approximate 35% decrease in enzymatic activity at 10 min, and little enzymatic difference was observed among ten IDH2 variants (R140G/Q/W and R172S/K/M/W/G/C/P) (Fig. 3B).

### IDH2 point mutations reduce glycolysis, increase dependency on mitochondrial oxidative phosphorylation, and decrease ROS levels

The levels of glucose, lactate, ATP, and ROS were evaluated 48 h post-transfection with WT or point IDH2 mutants in the 293T cell line under normal and hypoxic conditions (Fig. 3C–F). Under normal conditions, compared to WT, the IDH2 point mutation at site R172 resulted in an ~ 25% increase in glucose levels (Fig. 3C) and an ~ 25% decrease in lactate levels, except for R172P (Fig. 3D), but not the mutation at site R140 (Fig. 3C,D). Furthermore, under hypoxic conditions, overexpression of ten IDH2 point mutations in 293T cells led to a ~ 50% reduction in lactate levels (Fig. 3D), and ~ 1.5 fold and three- to six fold increases in glucose and ATP levels, respectively (Fig. 3C,E). In addition to R172P, other IDH2 point mutants reduced ROS levels by 40–60% under normal conditions, compared to WT (Fig. 3F). However, under hypoxic conditions, overexpression of IDH2 R140Q and R172P markedly decreased ROS levels, R140G, R172G, and R172C variants mildly reduced ROS, whereas other point mutants (R140W, R172/S/K/W/M/) minimally affected ROS levels, compared to WT (Fig. S1).

### IDH2 point mutations show anti-proliferative effects through inhibition of PI3K/AKT signaling

**Figure 4:**
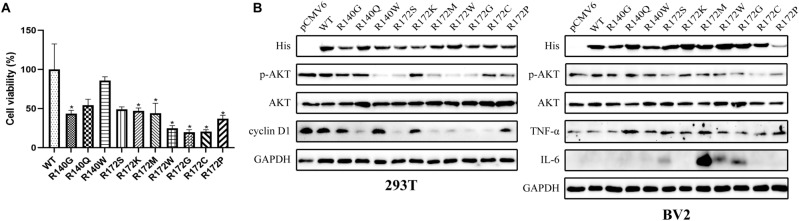
(**A**) Cell viability was evaluated by MTT assay in 293T at day 6 post-transfection with *IDH2* WT and mutation constructs. Data were normalized to WT and represent the mean values (± s.d.) of triplicate cultures. All the assays were performed from triplicate experiments. Statistically significant differences between WT and mutants are presented as *p < 0.05. (**B**) Immunoblotting was used to evaluate the expression of IDH2 (His), p-AKT, AKT, and cyclin D1 in the 293T cell line or IDH2 (His), p-AKT, AKT, TNFα, and IL6 in the BV2 cell line at 48 h post-transfection with *IDH2* WT or point mutation constructs. GAPDH stain is staining was used as a loading control.

Overexpression of ten IDH2 point mutants (except for R140W) in 293T cells reduced ∼50–70% in cell viability at 6 days after transfection with mutation constructs, compared with WT control (Fig. 4A). Immunoblotting evaluated the expression of p-AKT, AKT, and cyclin D1 in 293 T cells and, p-AKT, AKT, TNFα, and IL-6 in BV2 cells 48 h post-transfection with *IDH2* WT or point mutation constructs. Compared to WT IDH2, IDH2 point mutations markedly inhibited the expression of p-AKT (R140/G/W, R172S/M/W/G/C/P) in 293T and BV2 cell lines, and cyclin D1 (R140Q, R172S, R172M, R172W, R172G, and R172C), but R140Q and R172K point mutations had little affect on p-AKT expression in both cell lines (Fig. 4B and Table 1). However, the ratio of p-AKT/AKT is significantly decreased in 293T and BV2 cell lines (except for R140Q and R172M in BV2) after overexpression of the ten point mutations (Table 1). Expression quantitations of IDH2 (His), p-AKT, AKT, the ratio of p-AKT/AKT, and *p-value* of p-AKT/AKT are shown in Table 1. Expression of the IDH2 point mutation induced the expression of TNF-α (R140Q/W or R172S/K/M/W/G/C/P) and IL-6 (IDH2 R712S/M/W/G) in the BV2 cell line (Fig. 4B).

**Table 1:** Expression quantitations of IDH2 (His), p-AKT, and AKT in 293T and BV2 cell lines at 48 h post-transfection with *IDH2* WT and point mutant constructs.

		pCMV6	WT	IDH2 mutations									
				R140G	R140Q	R140W	R172S	R172K	R172M	R172W	R172G	R172C	R172P
293 T	His (IDH2)	0	1.00	0.53	0.98	0.94	0.60	0.56	0.90	1.07	0.63	0.75	0.67
	p-AKT	1.87	1.00	0.81	1.02	0.11	0.21	1.01	0.36	0.11	0.17	0.73	0.62
	AKT	1.74	1.00	1.22	1.97	1.88	1.73	1.71	2.23	1.70	1.73	2.06	2.29
	p-AKT/AKT	1.08	1.00	0.67****	0.52****	0.06****	0.12****	0.59****	0.16****	0.06****	0.10****	0.35****	0.27****
BV2	His (IDH2)	0	1.00	0.55	0.96	0.72	1.05	1.45	0.93	1.11	1.03	0.64	0.17
	p-AKT	0.92	1.00	0.62	0.92	0.79	0.49	0.85	0.60	0.56	0.42	0.17	0.34
	AKT	0.78	1.00	0.72	0.66	0.90	1.01	1.05	0.60	0.87	1.14	0.65	0.48
	p-AKT/AKT	1.17	1.00	0.86****	1.40****	0.87****	0.48****	0.81****	1.00	0.65****	0.37****	0.27****	0.72****

*p < 0.05, **p < 0.01, ***p < 0.001, and ****p < 0.0001.

### Point mutations in IDH2 decrease protein stability

**Figure 5:**
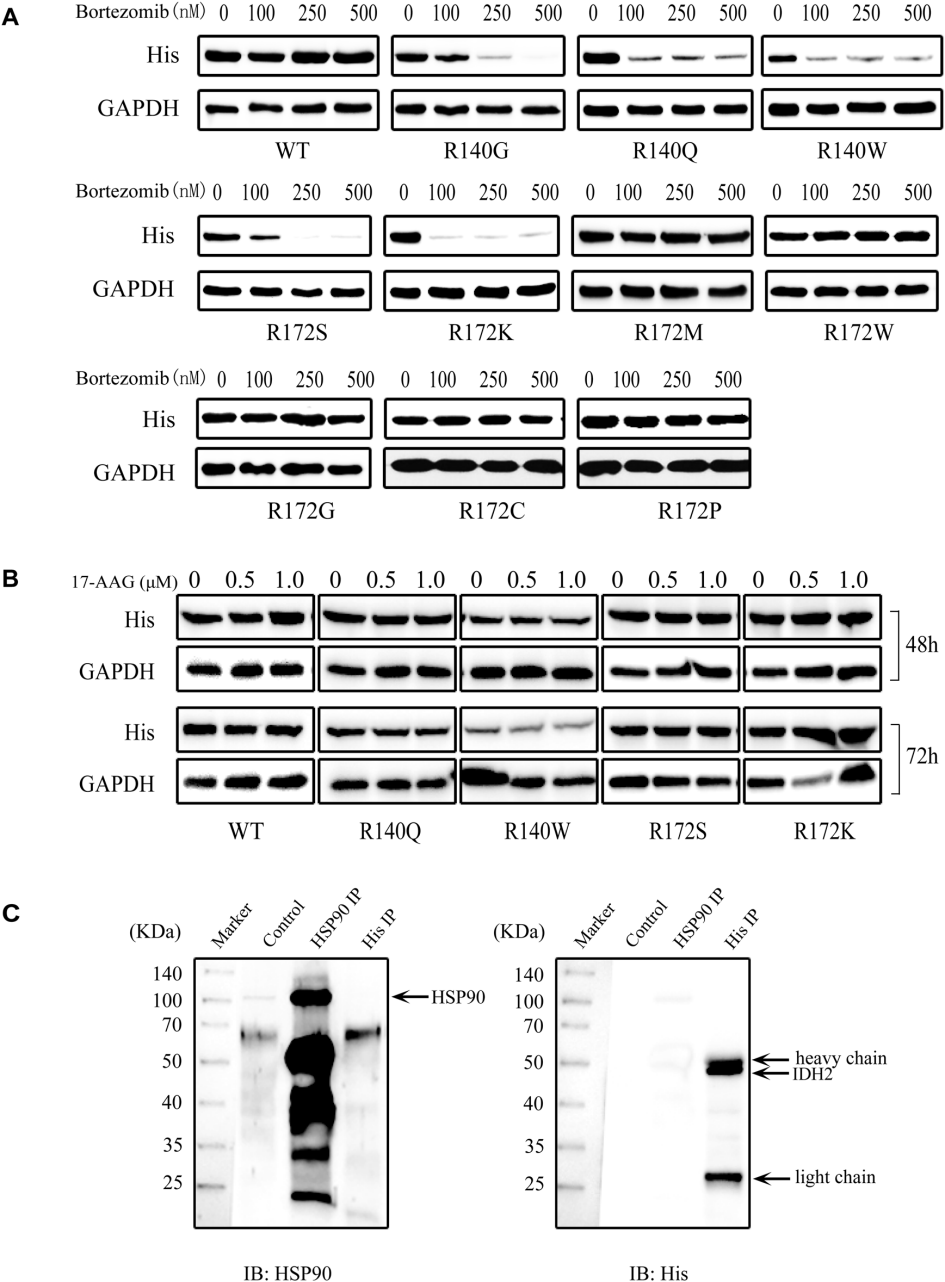
The stability of overexpressed IDH2 protein was evaluated after inhibition of the 20S proteasome (**A**) and HSP90 (**B**). Immunoblotting evaluation of IDH2 expression in 293T cells at 48 h post-transfection and then treated with bortezomib (100 nM, 250 nM, and 500 nM) for 72 h or 17-AAG (0.5 µM and 1 µM) for 48 or 72 h. GAPDH staining was used as a loading control. (**C**) The interaction of HSP90 and IDH2 was evaluated by HSP90 and His immunoprecipitation followed by His and HSP90 immunoblotting at 48 h post-transfection with the WT *IDH2* construct in 293T cells. The normal rabbit IgG immunoprecipitation is a negative control.

Immunoblotting evaluated the association of point mutations in IDH2 with protein stability in HEK293T cells 48 h post-transfection after treatment with different concentrations of the ubiquitin-tagged protein inhibitor bortezomib (100 nM, 250 nM, and 500 nM) for 72 h. Treatment with bortezomib markedly inhibited the expression of the IDH2 point mutation, including R140G, R140Q, R140W, R172S and R172K, but it barely affected the expression of IDH2 WT and mutants (R172M, R172W, R172G, R172C and R172P) (Fig. 5A).

Heat shock protein 90 (HSP90), a molecular chaperone, optimizes and maintains folding and localization for many client proteins and prevents their proteasomal degradation^19^. Inhibition of HSP90 by 17-AAG generally results in the degradation of client proteins through the ubiquitin–proteasome pathway. Thus, the stability of IDH2 proteins overexpressed in 293T cells was also evaluated by immunoblotting after treatment with the HSP90 inhibitor 17-AAG for 48 or 72 h (Fig. 5B). Compared to WT IDH2 expression, HSP90 inhibition had little effect on the expression of the ten point mutants.

The IDH2-HSP90 interaction was further investigated in the 293T cell line by HSP90 and His immunoprecipitation at 48 h post-transfection with the *IDH2* WT construct followed by His and HSP90 immunostaining (Fig. 5C). HSP90 co-IP did not reveal a dominant IDH2 50 kDa band; meanwhile, His co-IP did not show any bands at the HSP90 90 kDa position, and HSP90 and His immunoprecipitations did not show an association between IDH2 and HSP90, which was confirmed by HSP90 immunostaining after His immunoprecipitation and His immunostaining after HSP90 immunoprecipitation (Fig. 5C). Consistent with the above results, treatment with 17-AAG weakly reduced the expression of both IDH2 point mutants and WT (Fig. 5B).

### Structural analysis of IDH2 mutants

**Figure 6:**
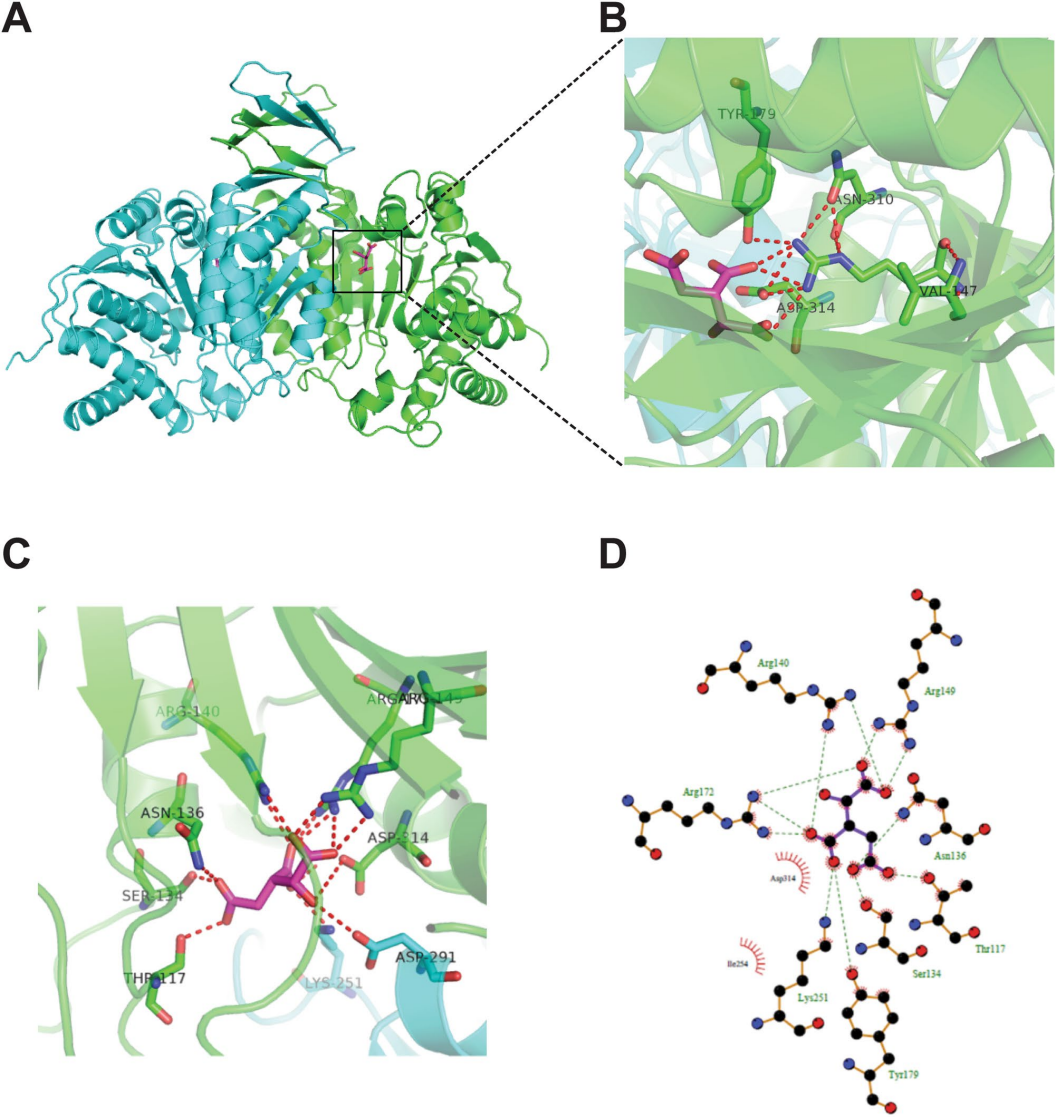
The effects of IDH2 mutations on the protein structure by bioinformation homology modeling. (**A**) Guided by the highly homologous PcIDH structure (PDB ID: 1LWD), a homology modeling structure of human wild-type IDH2 was constructed. (**B**) The interactions of R172 in the active site of human IDH2 and surrounding amino acids located on the surface of dimers were modeled, a dash line from (**A**) to (**B**), indicating the zooming part. The interactions of isocitrate and surrounding amino acids are shown. Hydrogen bonds (dashed line, **C**) and hydrophobic bonds (eyelashes, **D**) are represented.

Because the crystal structure of human IDH2 has not been determined, a homology model for human IDH2 was generated based on IDH2 from the published PcIDH structure (PDB ID: 1LWD)^6^, which shares 97% sequence identity with hIDH2 (Fig. S2 and Fig. 6A). As shown in Fig. 6B–D, both R140 and R172 of IDH2 located in the enzyme active sites play crucial roles in the interaction of the substrate isocitrate with the surrounding residues. The interactions of R172 and the surrounding amino acids located on the surface of the dimers were modeled (Fig. 6C,D). The R140 and R172 mutations led to the breaking of the hydrogen bonds with the surrounding residues and dimer formation, thereby decreasing the protein stability and its enzymatic activity.

## Discussion

IDH2 mutations are frequently found in glioma, chondrosarcoma, and acute myeloid leukemia and mainly occur in the active-site arginine residues of R140/R172^8–11^. Mutated IDH2 promotes cancer progression through metabolic reprogramming and epigenetic deregulation of gene expression. These mutations result in a product conversion from α-KG to 2-HG, which plays a pivotal role in the regulation of methylation, metabolism, the PI3K/AKT/mTOR signaling pathway and protein stability^20^. To date, the exact role of IDH2 mutations in carcinogenesis is not completely understood. Associations among the structure, function and stability of IDH2 mutants have rarely been reported, and these parameters are closely related to the occurrence and proliferation of cancer. Therefore, ten pathogenic IDH2 point mutants (R140G/Q/W and R172S/K/M/W/G/C/P) (Fig. 1) were generated and expressed in HEK293T and BV2 cell lines, and their enzymatic activity (Fig. 2), glucose, lactate, ATP, and ROS levels (Fig. 3 and Fig. S1), expression of pAKT, cyclin D1, TNFα, and IL6 (Fig. 4), and protein stability with 26S proteasome inhibition or HSP90 inhibition (Fig. 5) were compared between the WT and mutants.

The current data showed that overexpression of different IDH2 point mutants markedly reduced enzymatic activity compared to IDH2 WT (Fig. 3A,B), which has been demonstrated by previous studies showing that IDH2 mutants lose the ability to switch from NADP^+^ to NADPH^15^. IDH2 mutations resulted in dysregulation of a set of cellular metabolisms, including an increase in glucose (Fig. 3D) and ATP (Fig. 3E) but a decrease in lactate and ROS levels under normal and hypoxic conditions (Fig. 3C,F, and Fig. S1). These findings are consistent with a previous report that IDH2 mutations reduce glycolysis by inhibition and downregulation of pyruvate dehydrogenase (PDH), which induces the accumulation of pyruvate and glucose^21,22^. Furthermore, as compared to WT, point IDH2 mutation increases cell dependency on mitochondrial oxidative phosphorylation and reduces glycolysis, specially under hypoxic conditions, evidencing that IDH2 mutation overexpression resulted in ~ three- to six fold increase of ATP production (Fig. 3E). Under hypoxic conditions, compared to the changes in glucose, lactate, and ATP levels between R140 and R172 mutants, R172 and R140 point mutations showed the similar effects during glycolysis and oxidative phosphorylation (Fig. 3C–E), indicating that these point mutants of IDH2 play key roles in during glycolysis, TCA, and mitochondrial oxidative phosphorylation.

Loss of NADPH deficiency because of IDH2 mutant activity slows down the conversion of oxidized glutathione (GSSG) to reduced glutathione (GSH), which is a major antioxidant against oxidative stress induced by reactive oxygen species (ROS), and this may lead to ROS accumulation and oxidative DNA damage to promote tumor cell growth^23,24^. However, the present data showed that overexpression of IDH2 point mutations, except for R172P, reduced ROS levels in 293T cells under normal conditions (Fig. 3F). Overexpression of IDH2 R140Q and R172P markedly decreased ROS levels, R140G, R172G, and R172C variants mildly reduced ROS under hypoxic conditions (Fig. S1), which is probably due to two reasons: (1) IDH2 mutations resulted in downregulation of glutamic acids, such as in glioma^25^; (2) IDH2 mutations reduced cell viability through inhibition of PI3K/AKT signaling and slowed cell metabolism in 293 T and BV2 cells (Figs. 3, 4 and Table 1), which is consistent with a recent report that suppression of IDH2 inactivated AKT intermediates^26^ and the mutated IDH1 inhibited PI3K/AKT signaling in human glioma^27^. The different functional characteristics of R172P are possibly due to the fact that the proline side chain is bonded to the backbone nitrogen atom and to the alpha carbon atom. This cyclic structure may destroy the secondary structure and greatly influences the protein architecture.

In addition, TNF-α and IL-6 have been found to promote tumor cell proliferation, invasion, metastasis and angiogenesis in cancers^28^. In BV2 cells, overexpression of IDH2 mutants resulted in upregulation of TNF-α and IL-6 (Fig. 4B). We speculate that the occurrence of tumors associated with IDH2 mutations may be closely related to inflammatory reactions, but the specific mechanisms need further investigation.

Furthermore, the investigation of IDH2 mutant stability contributes to deeply understanding the molecular mechanisms in tumorigenesis and exploring new therapeutic strategies. Bortezomib is a dipeptide boronic acid inhibitor of the 26S proteasome that is FDA-approved for the treatment of multiple myeloma and mantle cell lymphoma^29–31^. In addition, HSP90, a chaperone protein, assists client protein folding and degradation of misfolding proteins. However, targeting HSP90 with 17-AAG shows anti-proliferative and pro-apoptotic effects through the degradation of mutated driver oncogenic proteins, such as EGFR in lung cancer^32^, HER2 in breast cancer^33^, and KIT or cyclin D1 in gastrointestinal stromal tumors^34,35^. Thus, changes in protein expression levels after inhibition of the 26S proteasome or HSP90 reflect IDH2 protein stability. In the present report, the stability of ten overexpressed point IDH2 mutants was evaluated by immunoblotting after treatment with bortezomib or 17-AAG (Fig. 5). Marked degradation of the IDH2 mutants, including R140G, R140Q, R140W, R172S and R172K, was observed post-treatment with bortezomib, whereas bortezomib treatment had little effect on the expression of WT and other mutants (R172M, R172W, R172G, R172C and R172P) (Fig. 5A), indicating that the protein stability of IDH2 mutants depends on arginine being mutated to other amino acids. However, HSP90 inhibition only slightly affected the expression of mutated IDH2 due to a lack of interaction between HSP90 and IDH2 (Fig. 5B,C). The homology model further revealed that IDH2 R140 and R172 are crucial amino acids acting on the surface of the dimer and surrounding amino acids, and different mutations of R140 and R172 led to the instability of the protein structure to varying degrees (Fig. 6), indicating that changes in the IDH2 conformation, dimerization, and protein stability appear to be associated with these point mutations. The study of the structural stability of IDH2 mutants will be of great significance in the clinical treatment of IDH2 mutation-induced diseases.

In conclusion, ten cancer-associated IDH2 point mutants (R140G/Q/W and R172S/K/M/W/G/C/P) have been demonstrated to accumulate 2-HG and promote tumor proliferation by altering a number of downstream cellular activities in the previous studies. The present data showed that there are some differences in protein conformational changes, stability, glycolysis, mitochondrial oxidative phosphorylation, and PI3K/AKT signaling among these IDH2 point mutations, specifically for the most common IDH2 point mutations R140Q and R172K in cancer cells, indicating that each IDH2 point mutation possibly plays a different biological role in tumorigenesis. However, this conception needs further investigation and analysis among these point mutants in detail. These novel findings provide important information for understanding the molecular mechanisms that underlie the pathogenesis of IDH2-mutated tumors.

## Supplementary Information

Supplementary Information.

## Data Availability

All data generated or analyzed during this study are included in this published article and its supplementary information files. Human IDH2 tertiary structure predictions generated and analysed during the current study are from the PcIDH structure (PDB ID: 1LWD) repository, https://www.ncbi.nlm.nih.gov/Structure/pdb/1LWD.
