# Supplementary material for: Effects of cancer-associated point mutations on the structure, function, and stability of isocitrate dehydrogenase 2
Source: Sci Rep. 2022 Nov 5;12:18830. doi: 10.1038/s41598-022-23659-y (PMC9637083; doi:10.1038/s41598-022-23659-y)
Supplement: Supplementary file 1 — Supplementary Information. [file 41598_2022_23659_MOESM1_ESM.pdf]

Table S1. Amplification primers of *IDH2* point mutants

Primer name	Primer sequences
WT *IDH2*-F	5'CCCAAGCTTATGGCCGGCTACCTGCGGGTCGTGC3'(*Hin*d III)
WT *IDH2*-R	5'CGACGCGTCTGCCTGCCCAGGGCTCTGTCCAGGT3'(*Mlu* I)
R140G-Fm	GGAACTATCGGGAACATCCTGGGGGG
R140G-Rm	CCAGGATGTTCCCGATAGTTCCATTGGG
R140Q-Fm	CAGAACATCCTGGGGGGGACTGTCTTC
R140Q-Rm	GAAGACAGTCCCCCCCAGGATGTTCTG
R140W-Fm	GGAACTATCTGGAACATCCTGGGGGGG
R140W-Rm	CCAGGATGTTCCAGATAGTTCCATTGGG
R172S-Fm	CACCATTGGCAGCCACGCCCAT
R172S-Rm	GGCGTGGCTGCCAATGGTGATGG
R172K-Fm	TCACCATTGGCAAGCACGCCCAT
R172K-Rm	GCGTGCTTGCCAATGGTGATGGG
R172M-Fm	ATCACCATTGGCATGCACGCCCAT
R172M-Rm	TGGGCGTGCATGCCAATGGTGAT
R172W-Fm	ACCATTGGCTGGCACGCCCAT
R172W-Rm	GCGTGCCAGCCAATGGTGATGG
R172G-Fm	CATCACCATTGGCGGGCACGC
R172G-Rm	GTGCCCGCCAATGGTGATGGG
R172C-Fm	ACCATTGGCTGCCACGCCCAT
R172C-Rm	GCGTGGCAGCCAATGGTGATGG
R172P-Fm	ACCATTGGCCCGCACGCCCAT
R172P-Rm	GCGTGCGGGCCAATGGTGATG

Figure S1

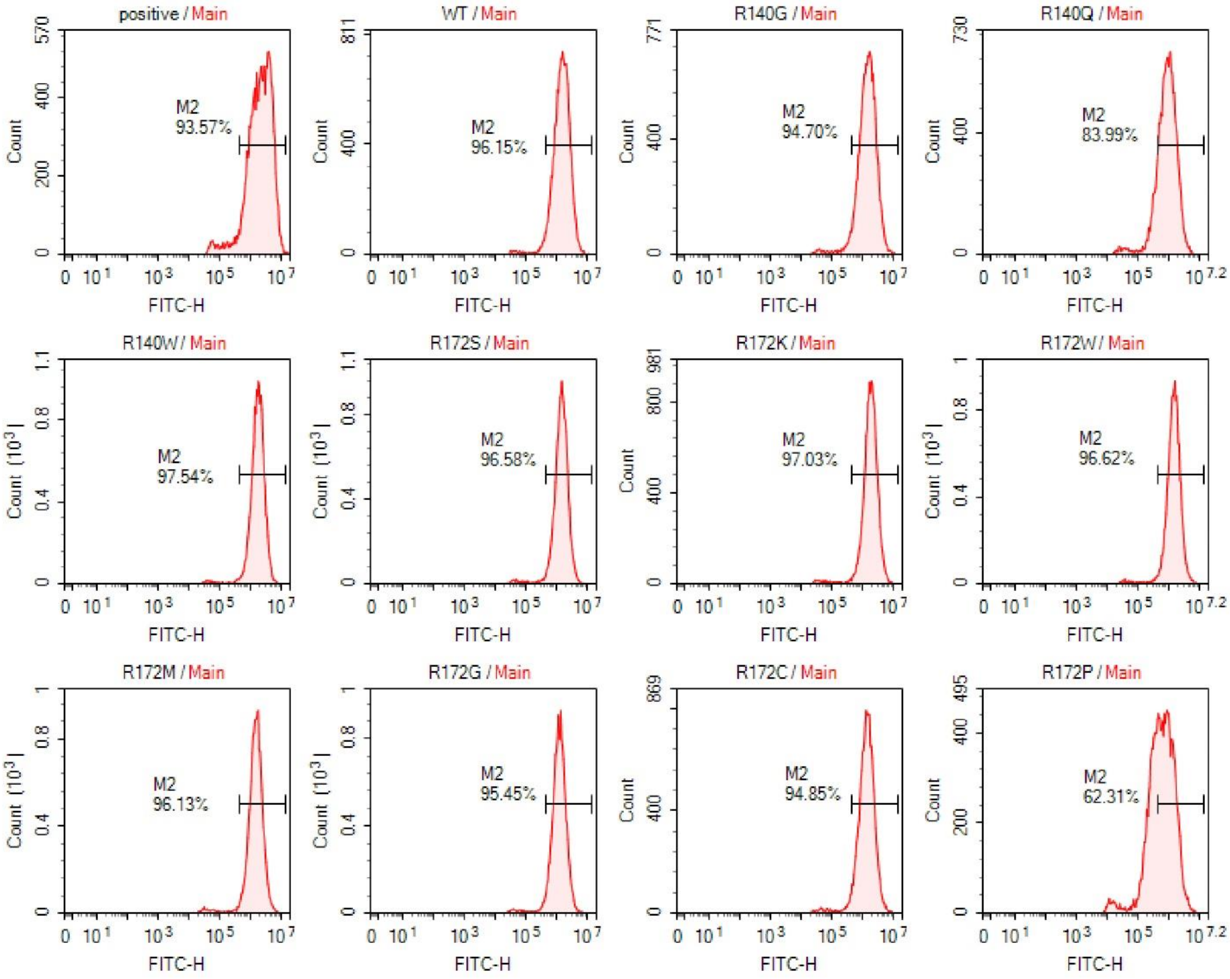

Figure S1. ROS levels were measured under hypoxic conditions (200 μM $CoCl_2$ treatment) using a Reactive Oxygen Species Assay Kit. The DCF fluorescence distribution of the cells was detected at FITC channel by a flow cytometry. The oxidative conversion of cell permeable DCFH-DA to DCF indicates changes in intracellular ROS levels.

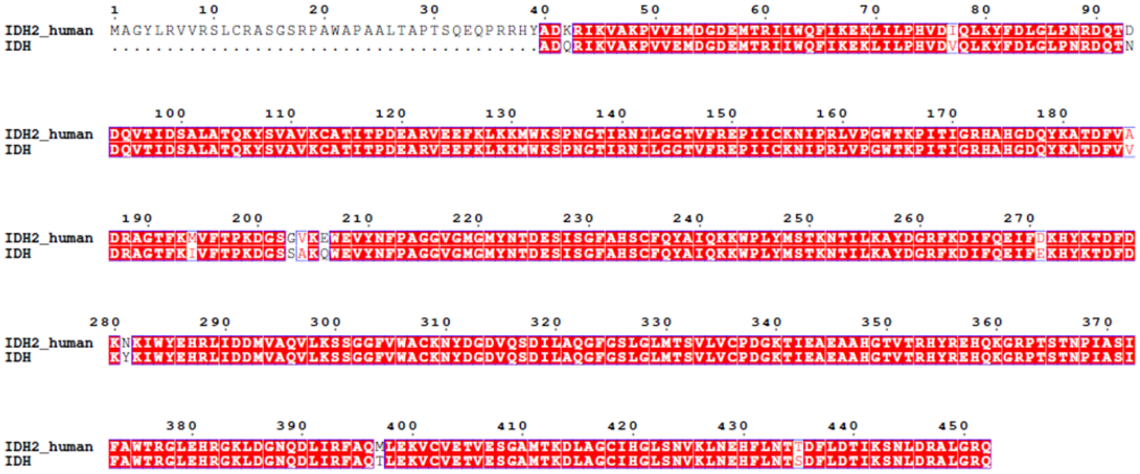

Figure S2. Sequence homology comparison between human IDH2 and porcine heart mitochondrial isocitrate dehydrogenase (PcIDH)

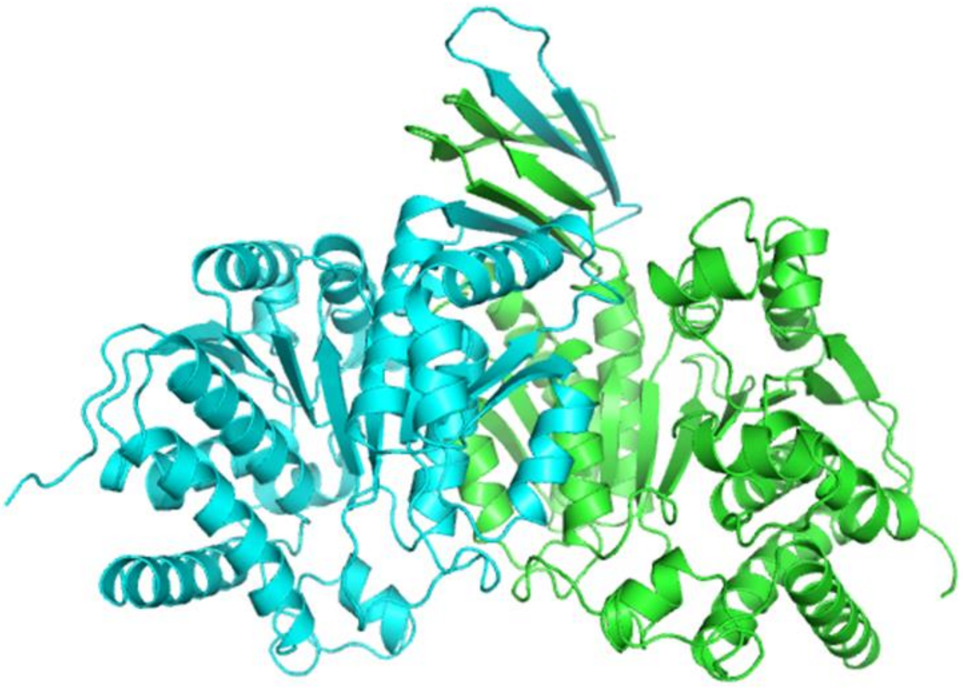

A homology model for human IDH2

IDH2 original western blots

Figure 2A

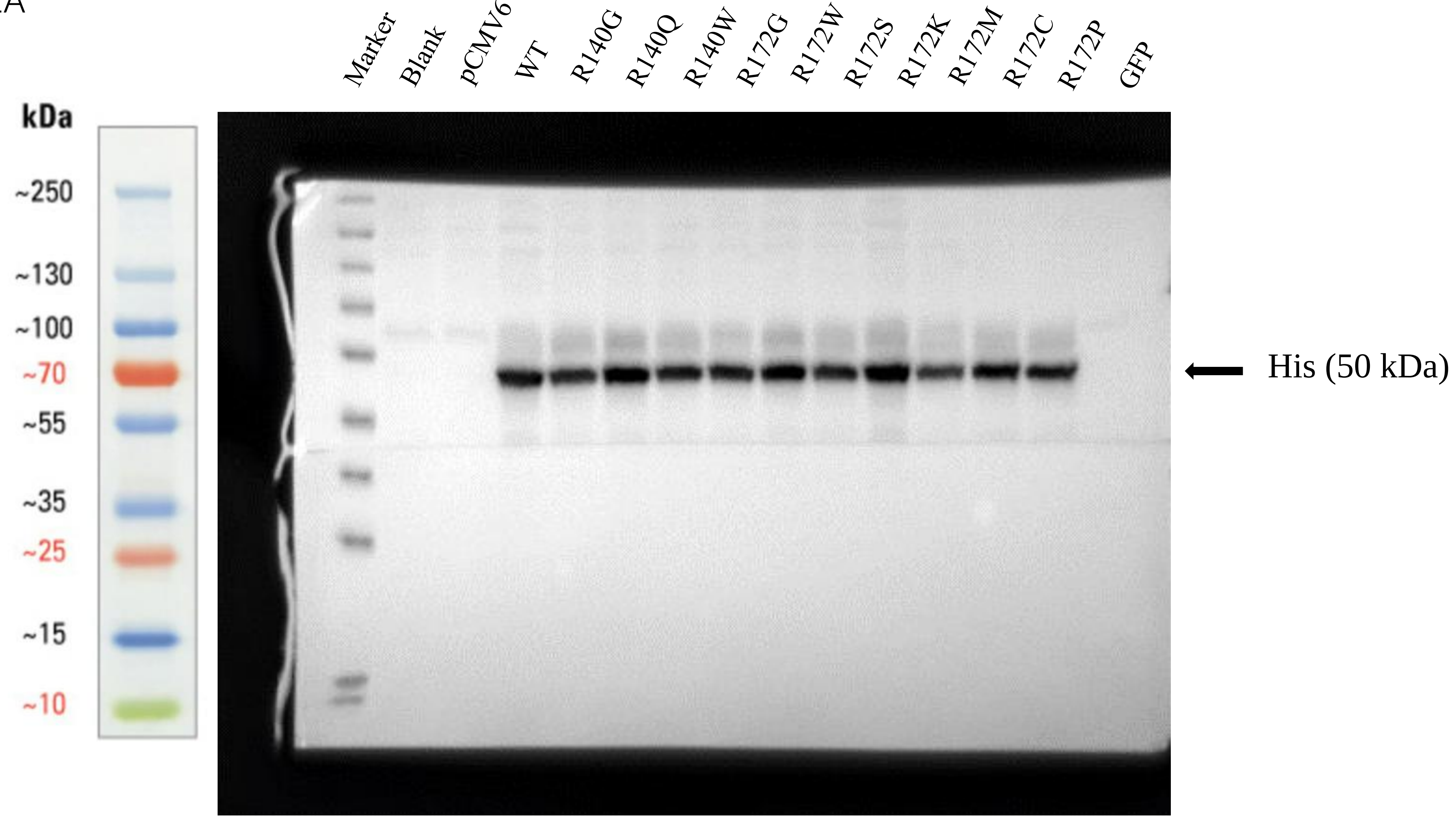

293T

Figure 2A

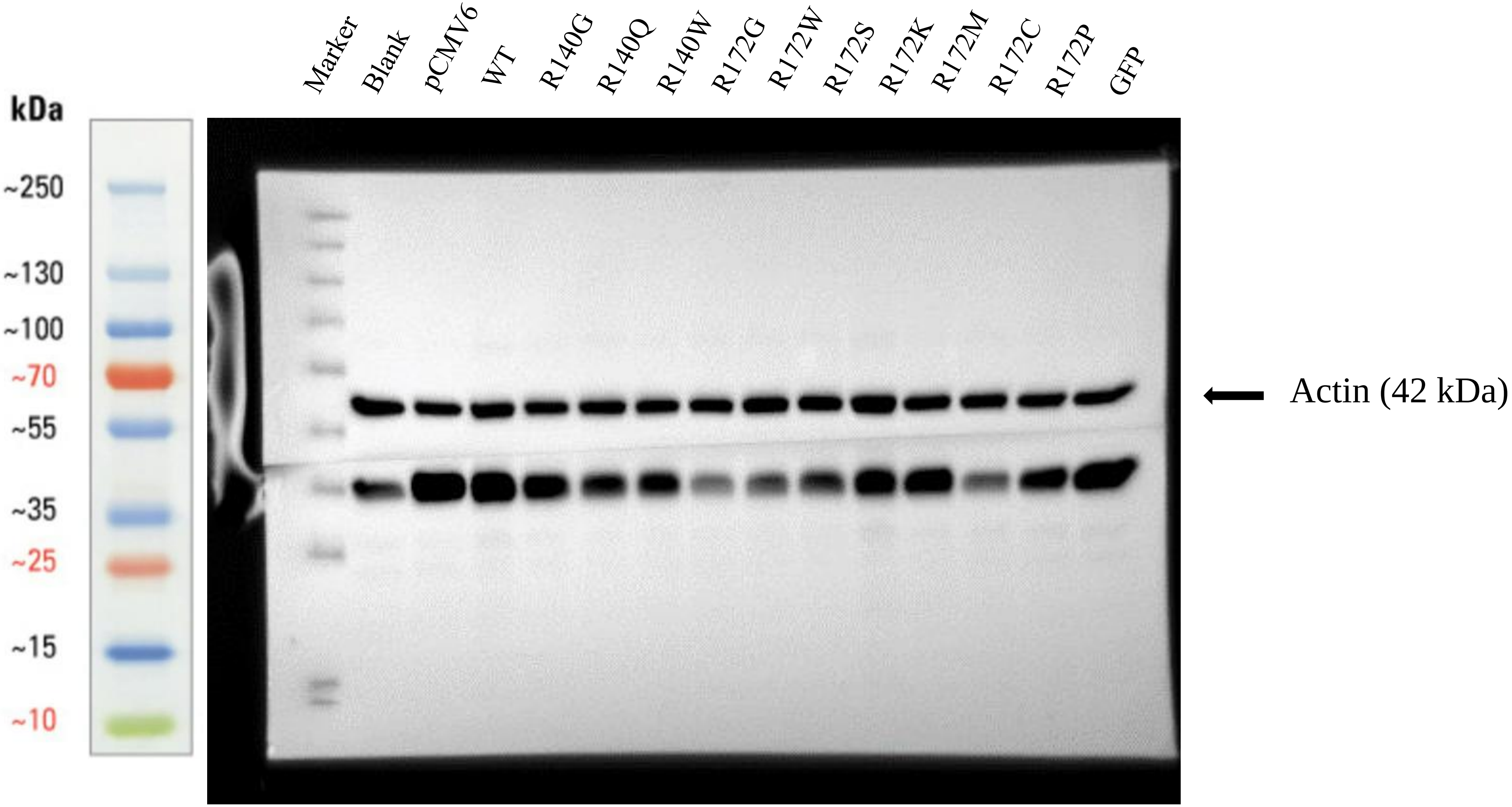

293T

Figure 2B

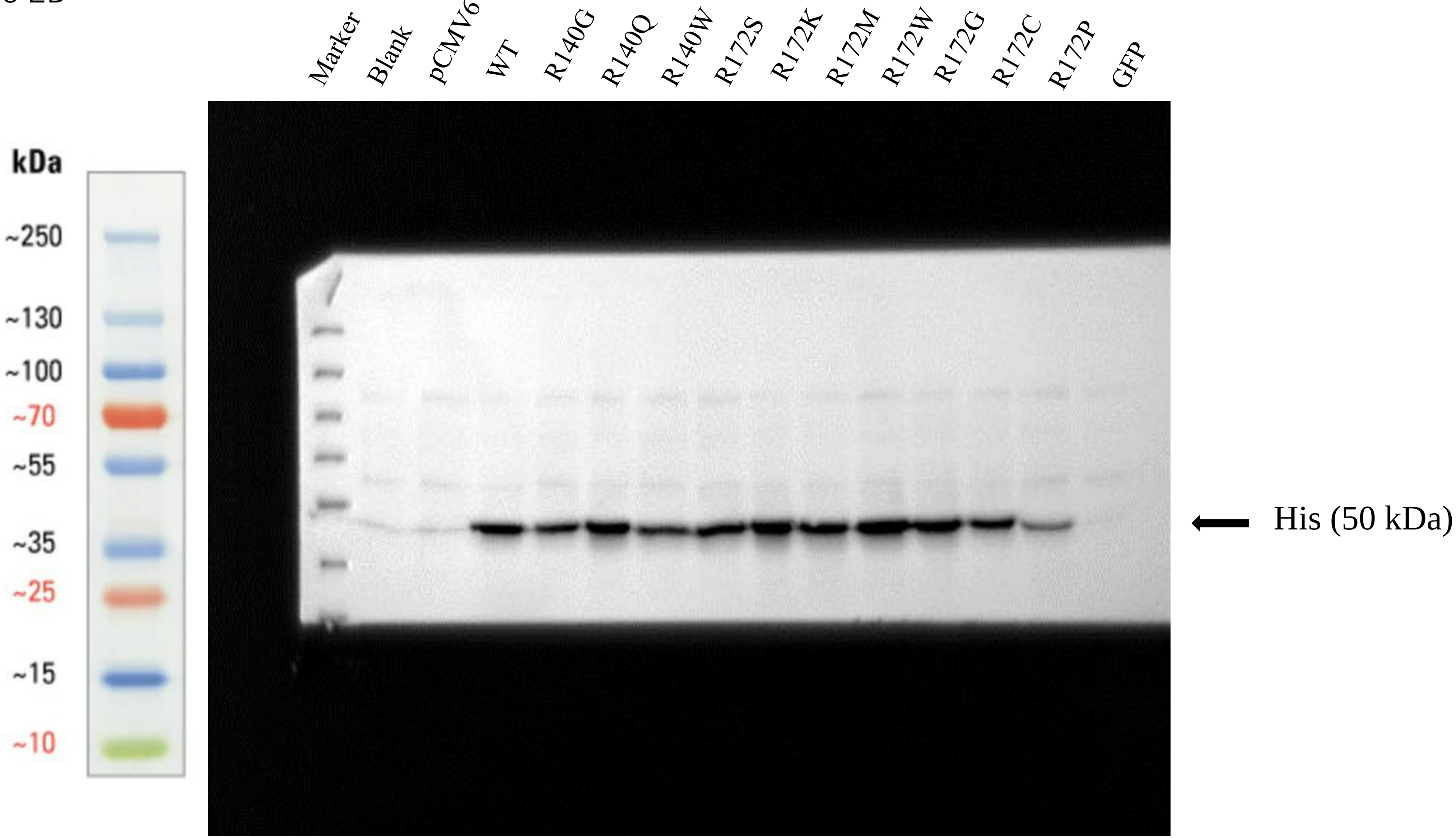

BV2

Figure 2B

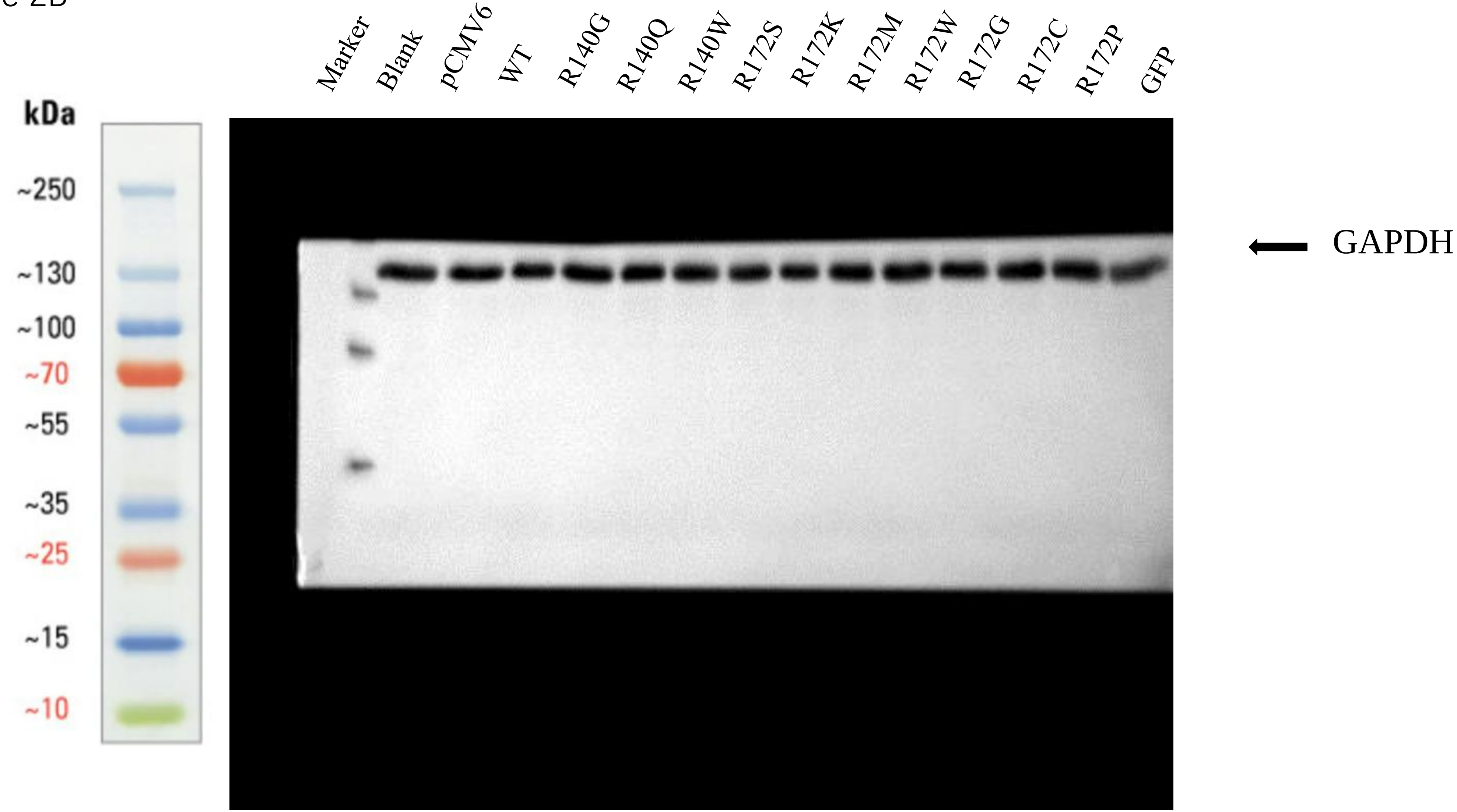

BV2

Figure 4A

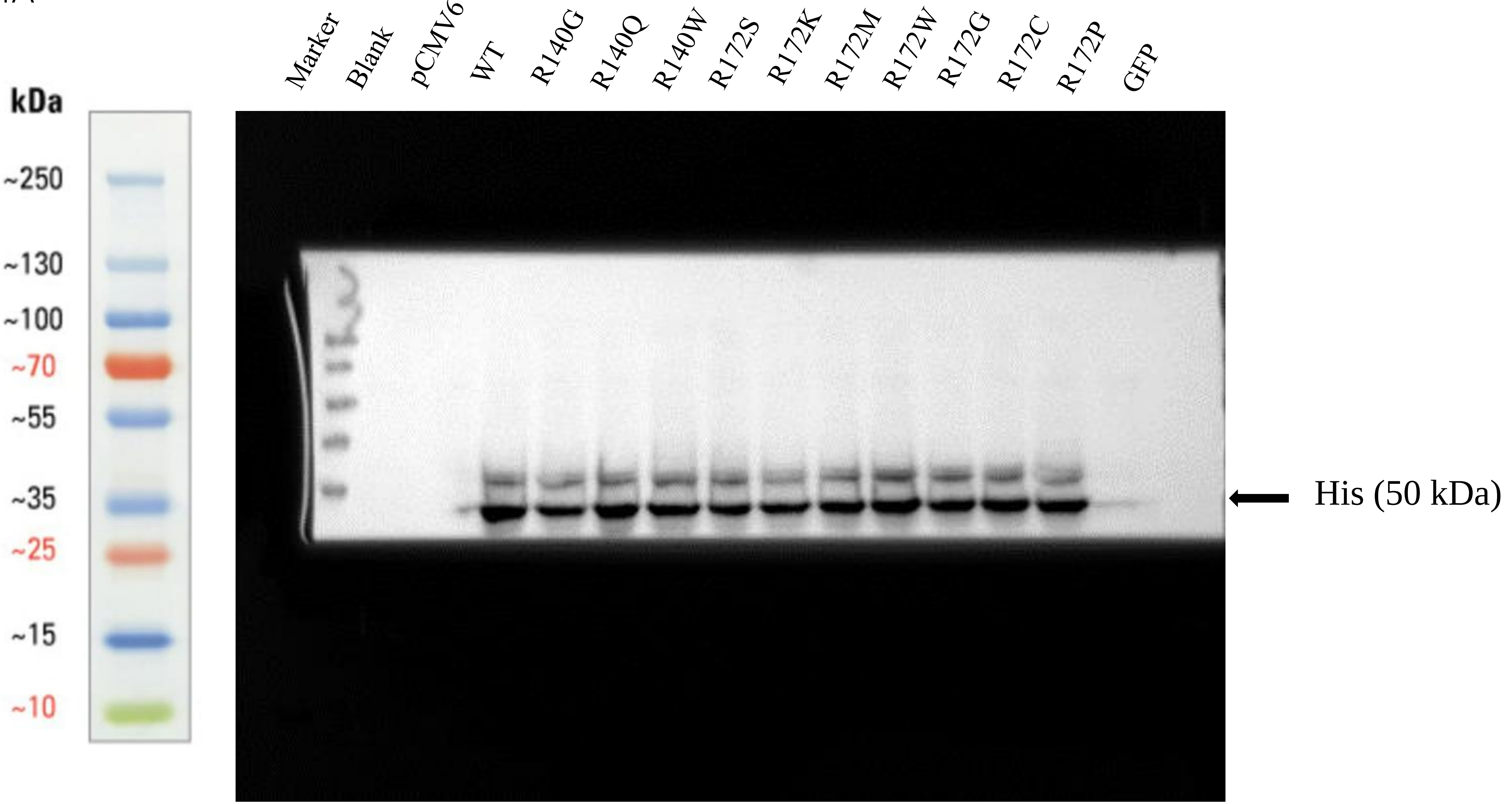

293T

Figure 4A

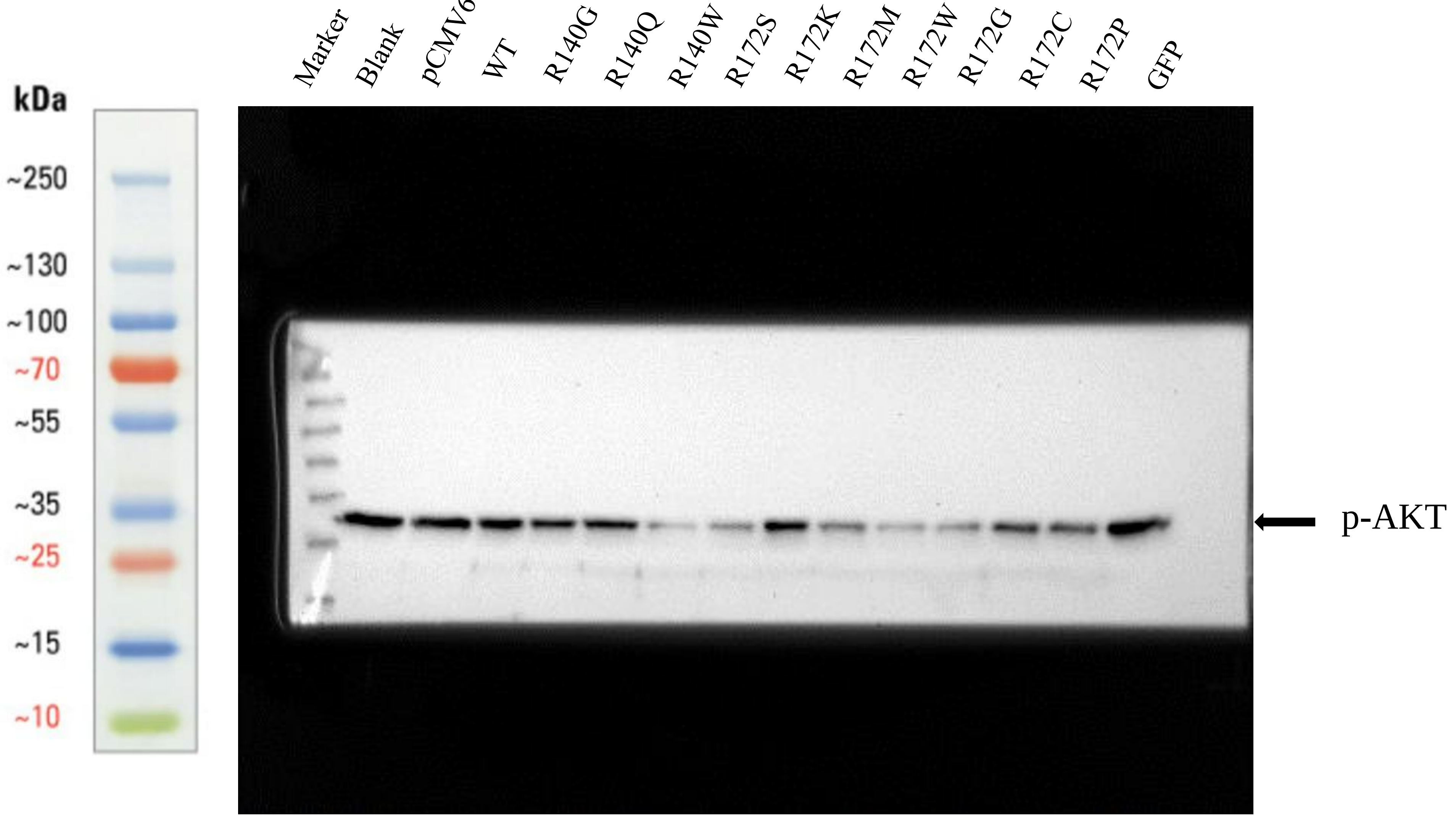

293T

Figure 4A

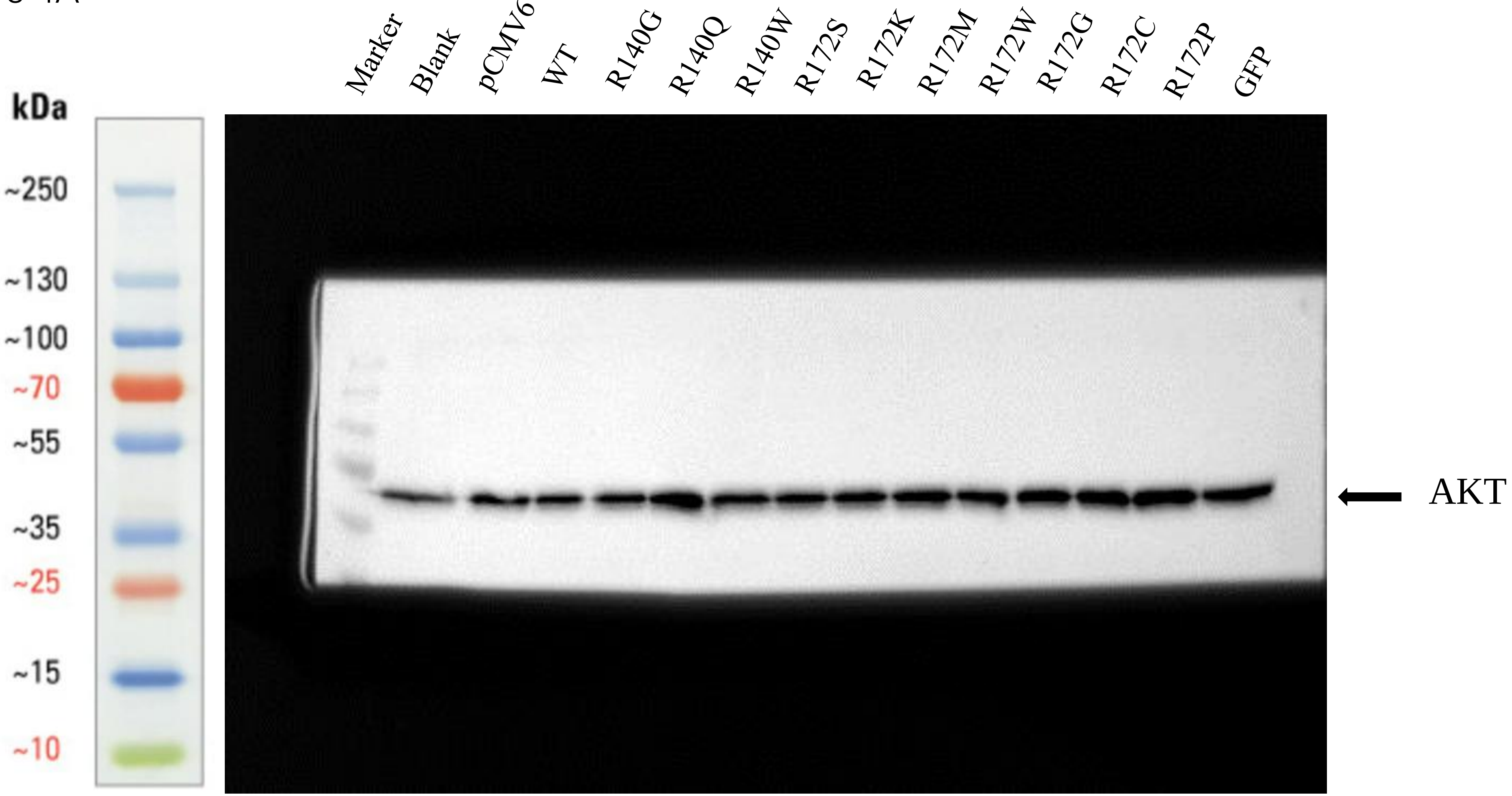

293T

Figure 4A

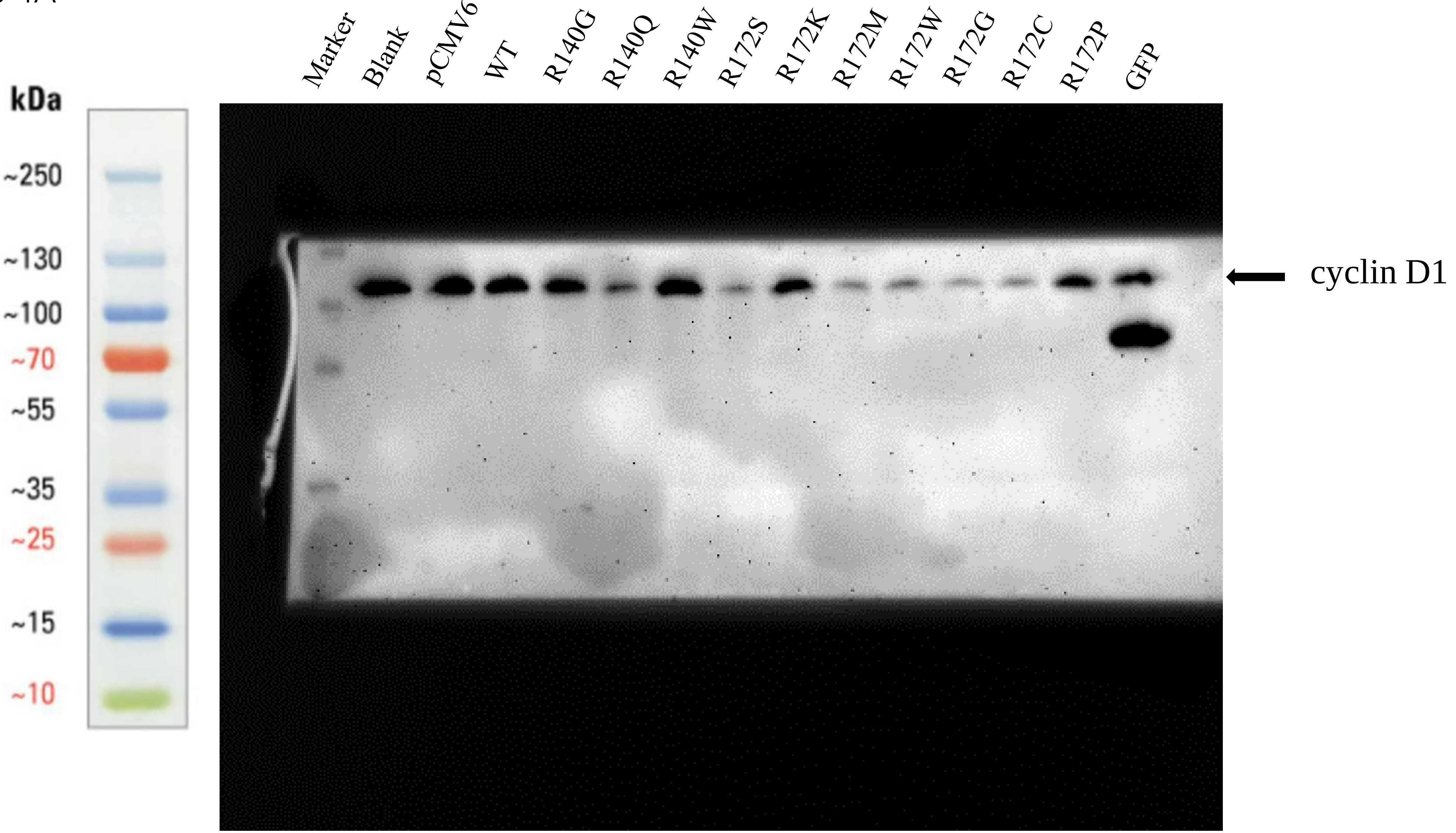

293T

Figure 4A

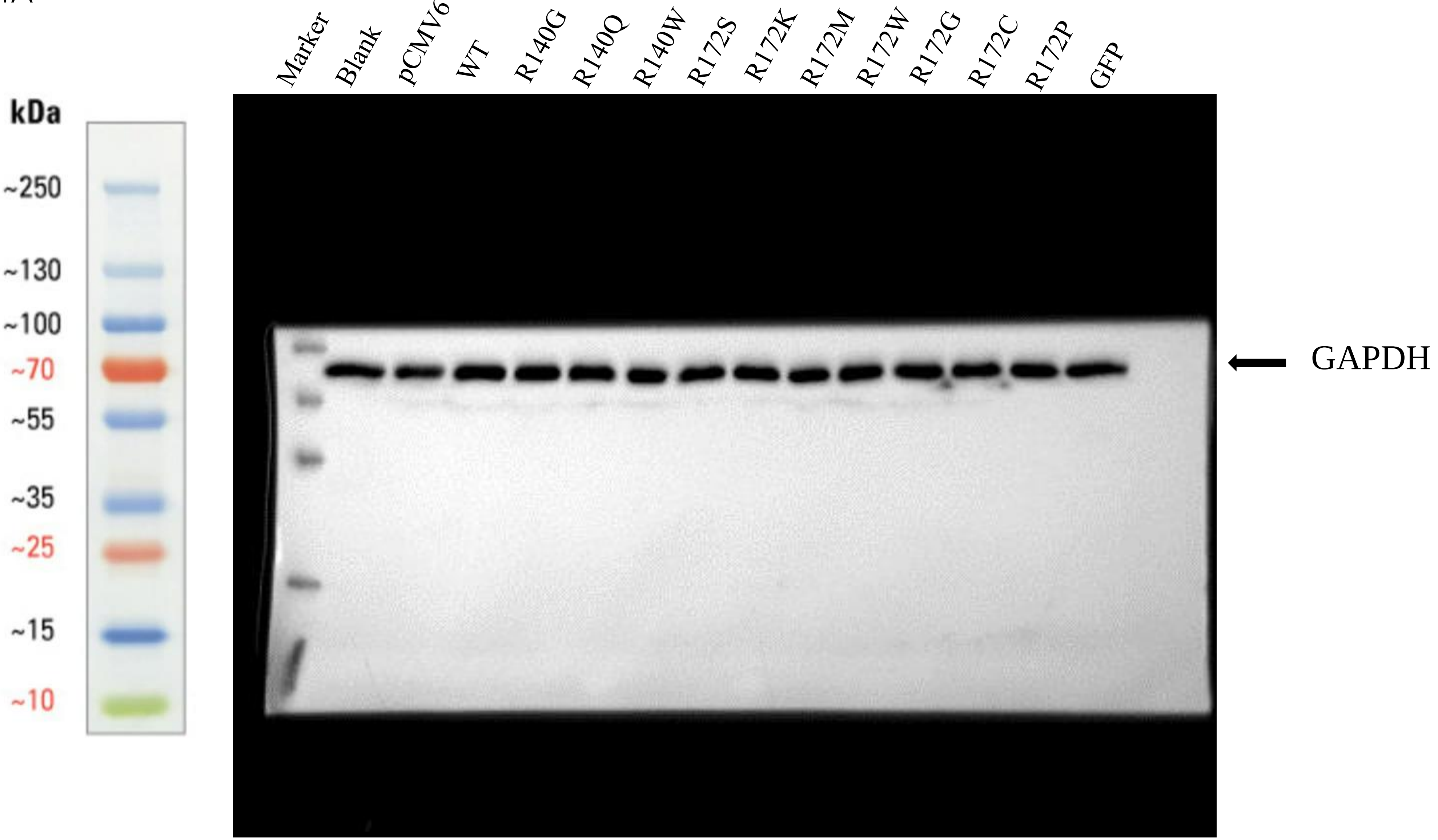

293T

Figure 4B

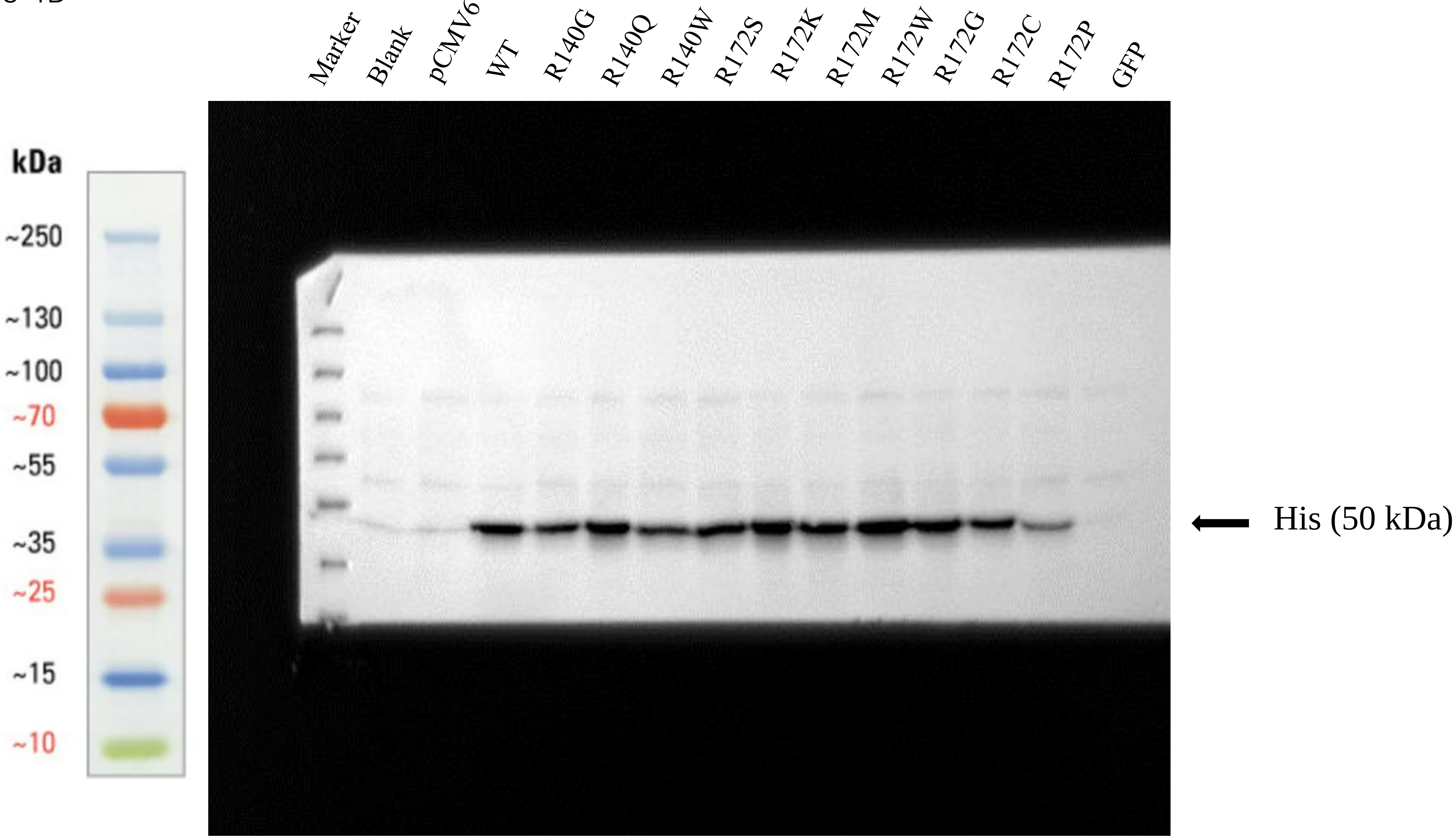

BV2

Figure 4B

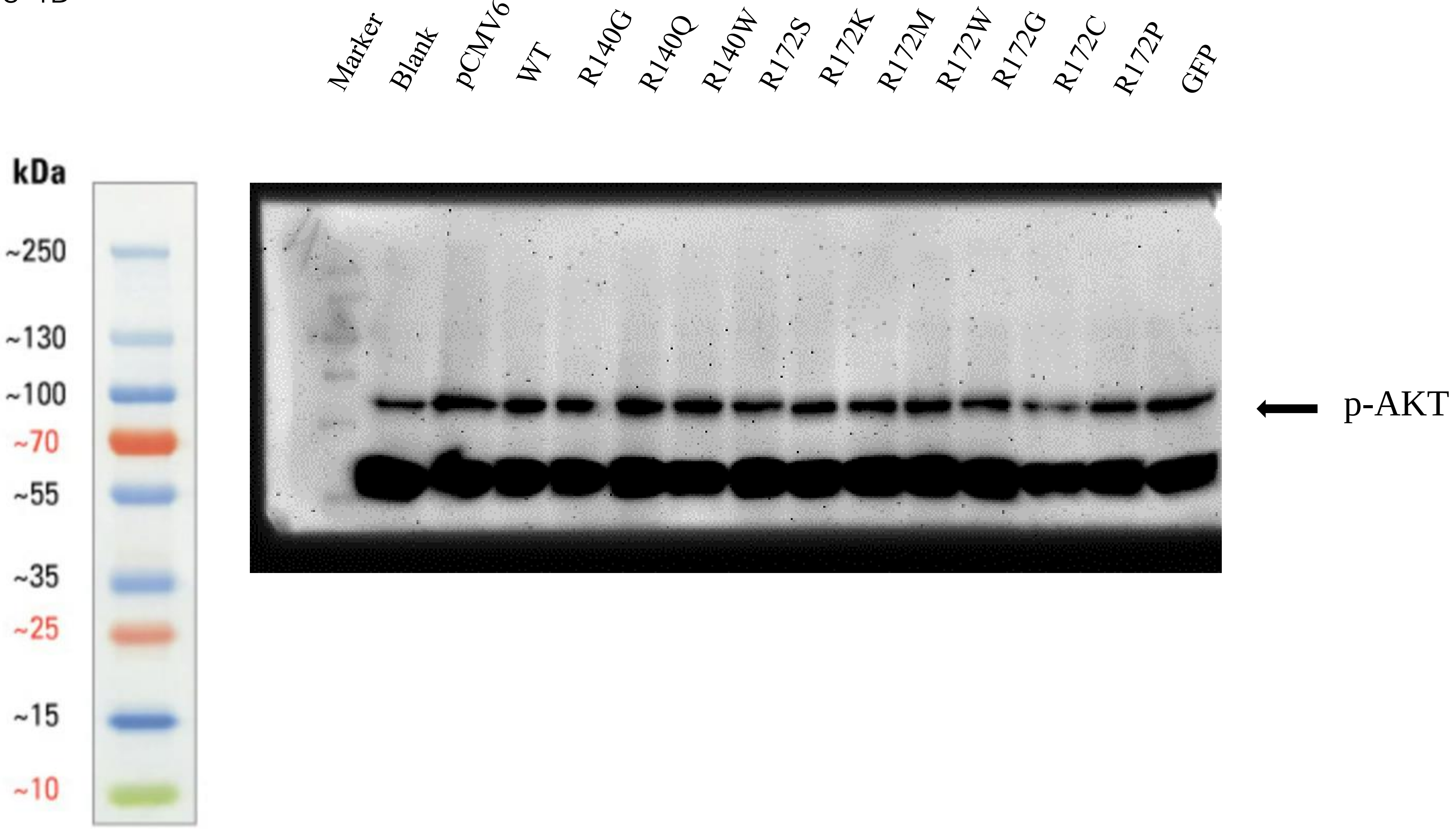

BV2

Figure 4B

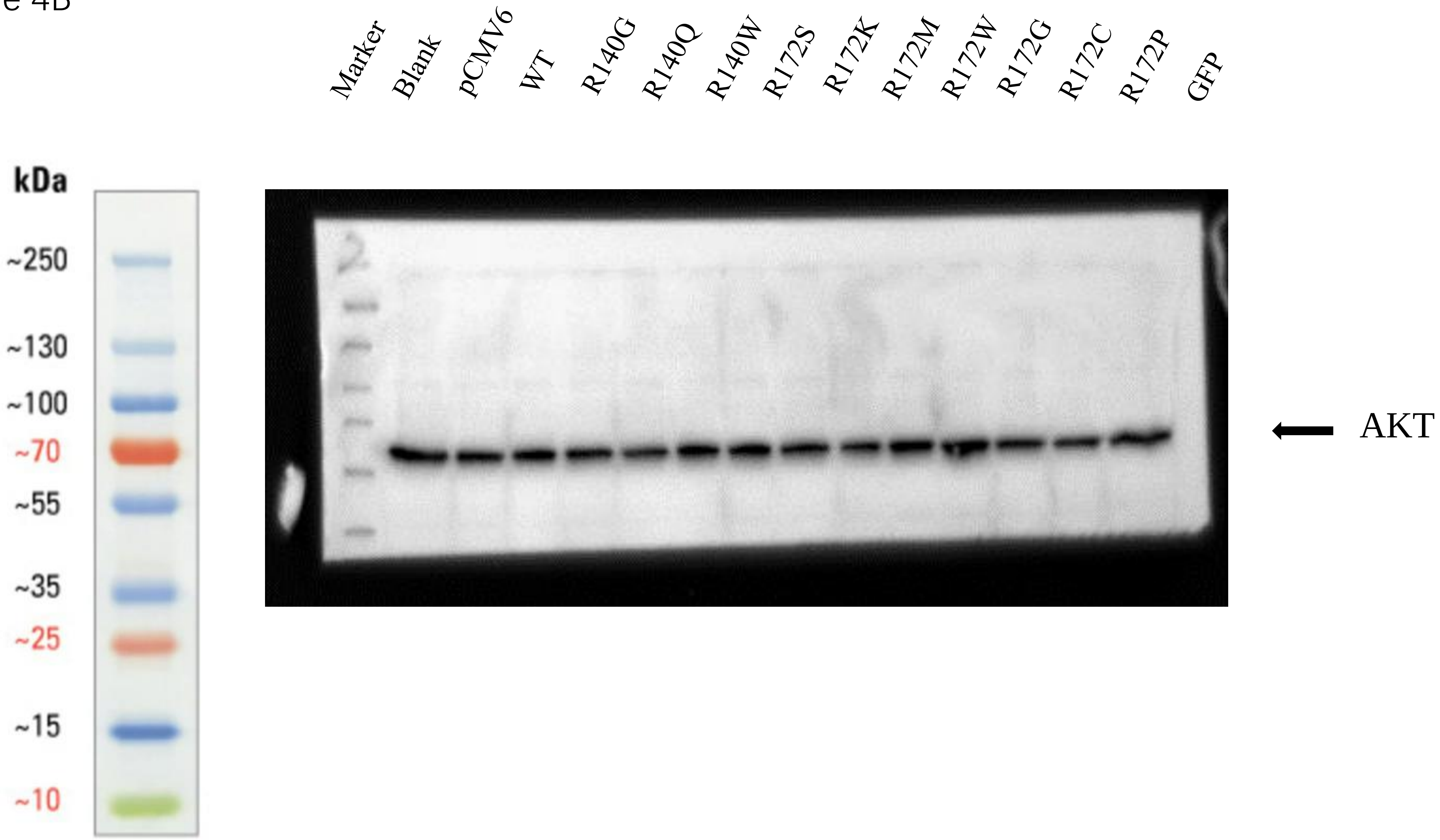

BV2

Figure 4B

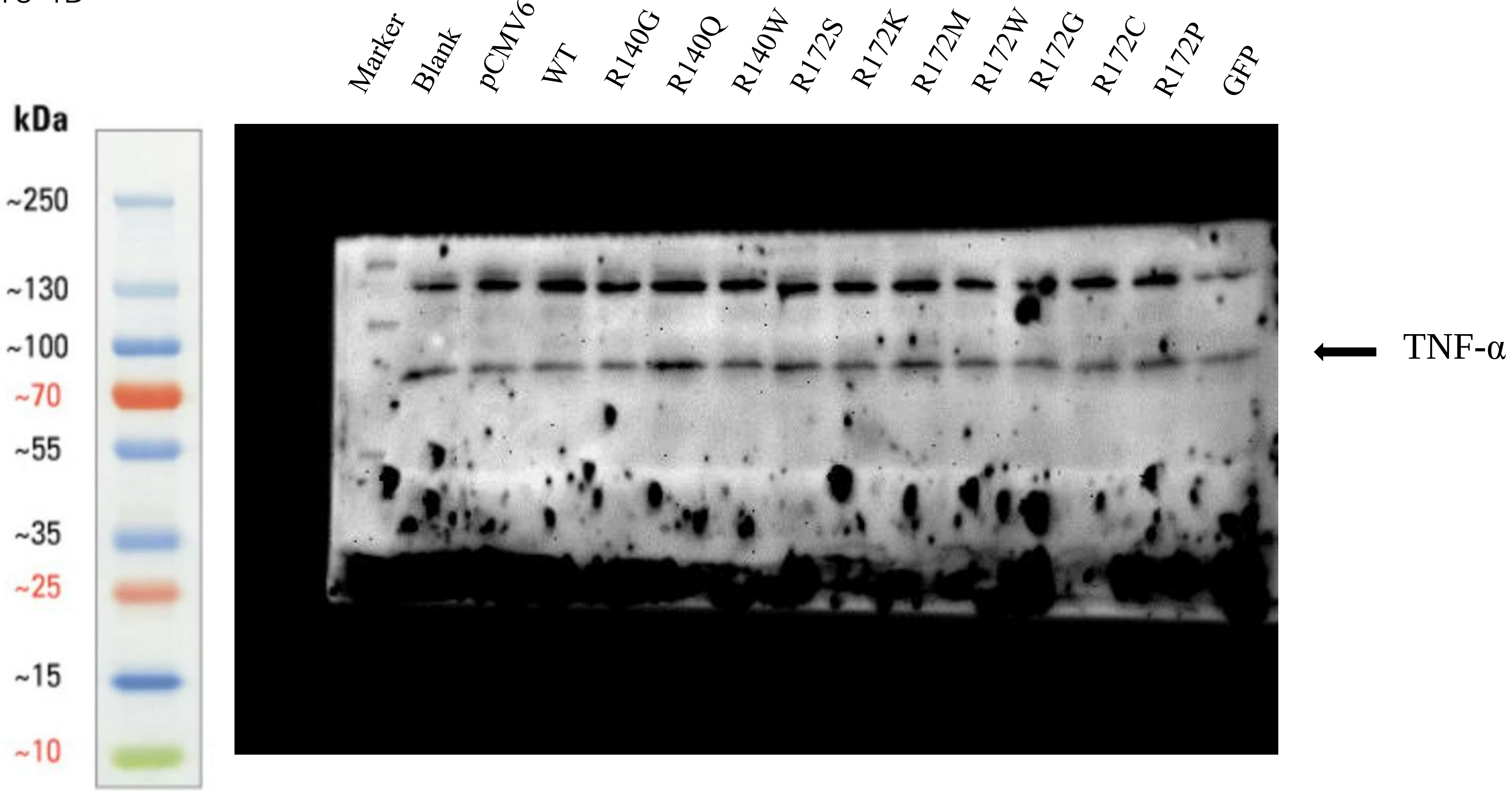

BV2

Figure 4B

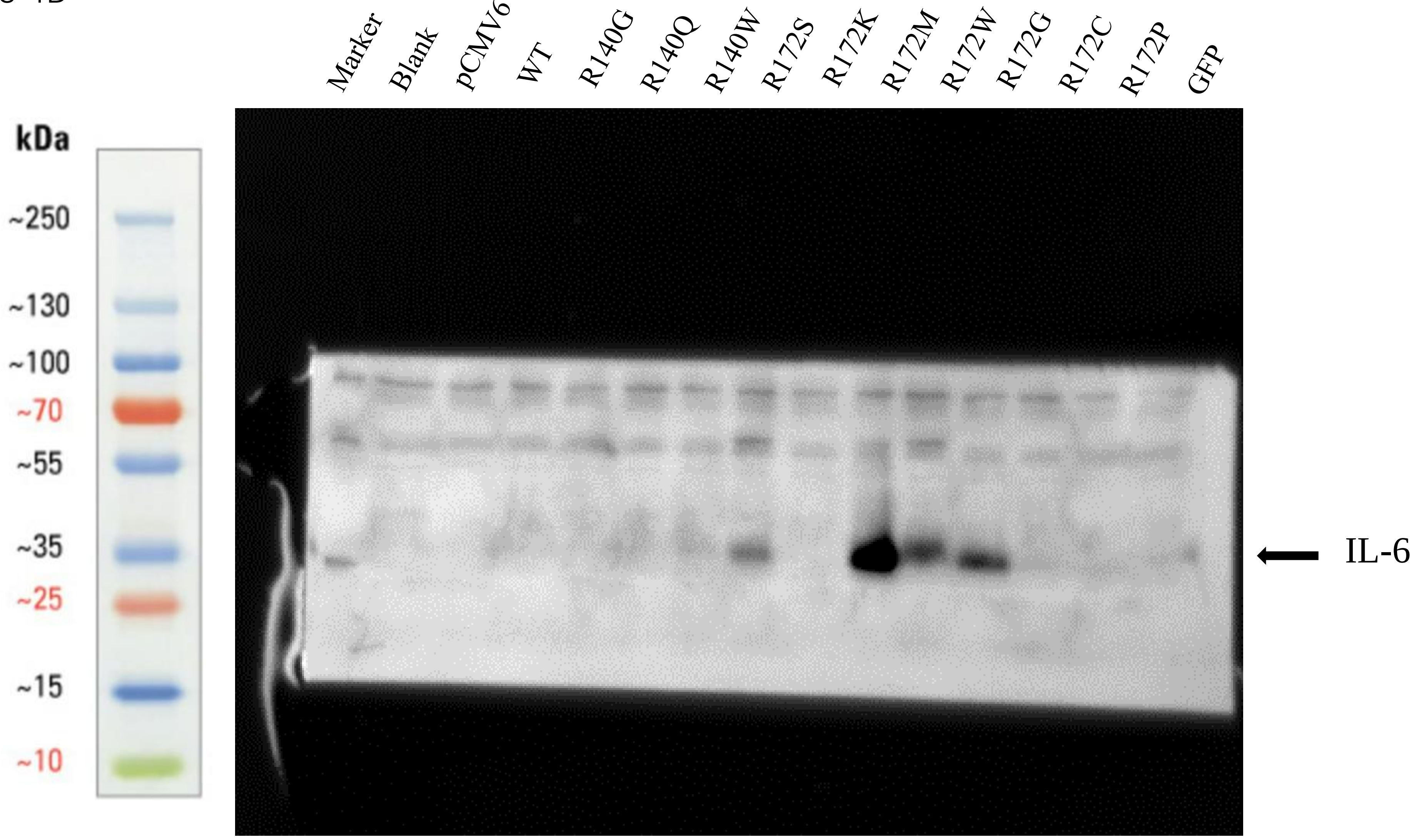

BV2

Figure 4B

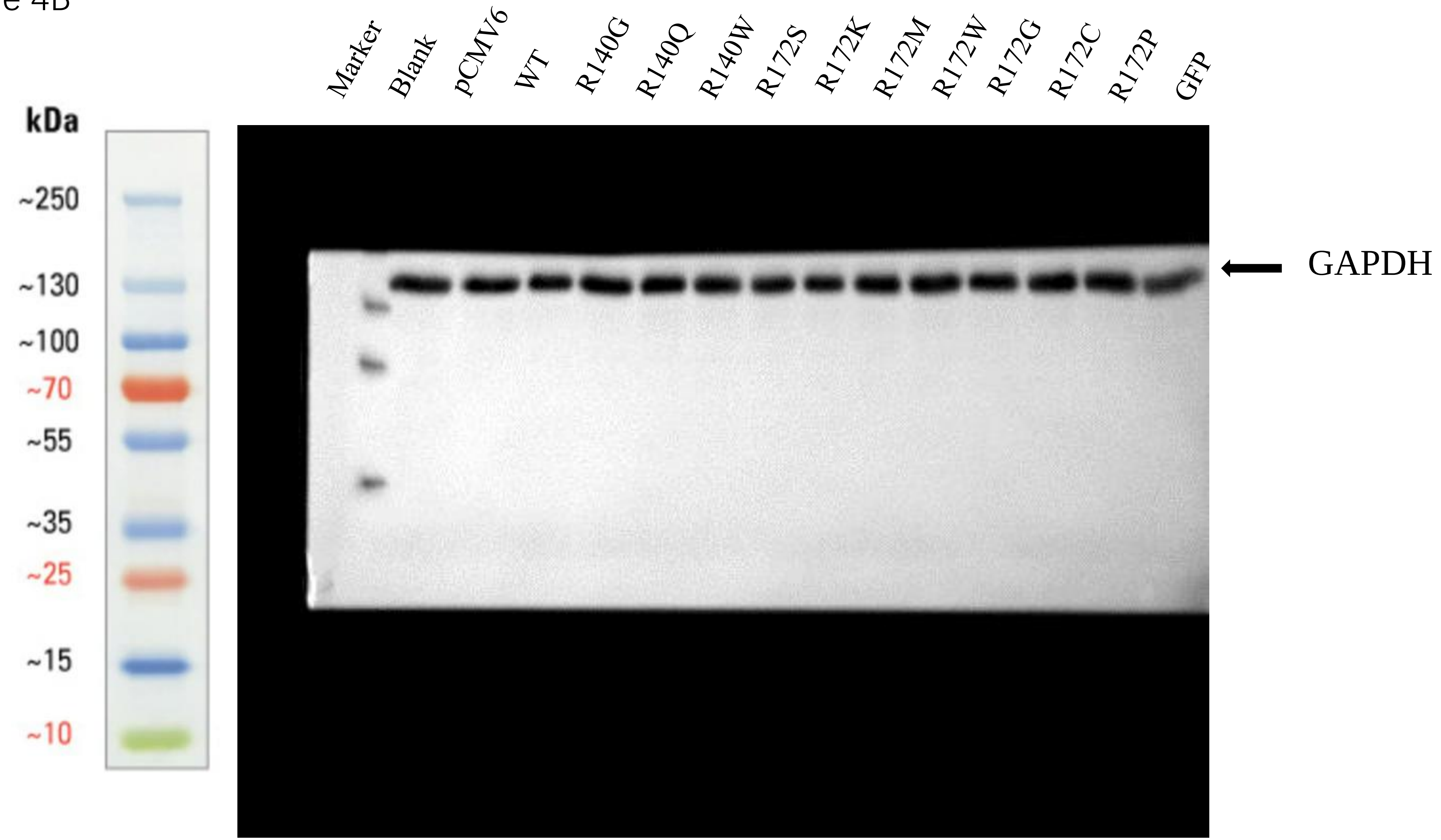

BV2

Figure 5A

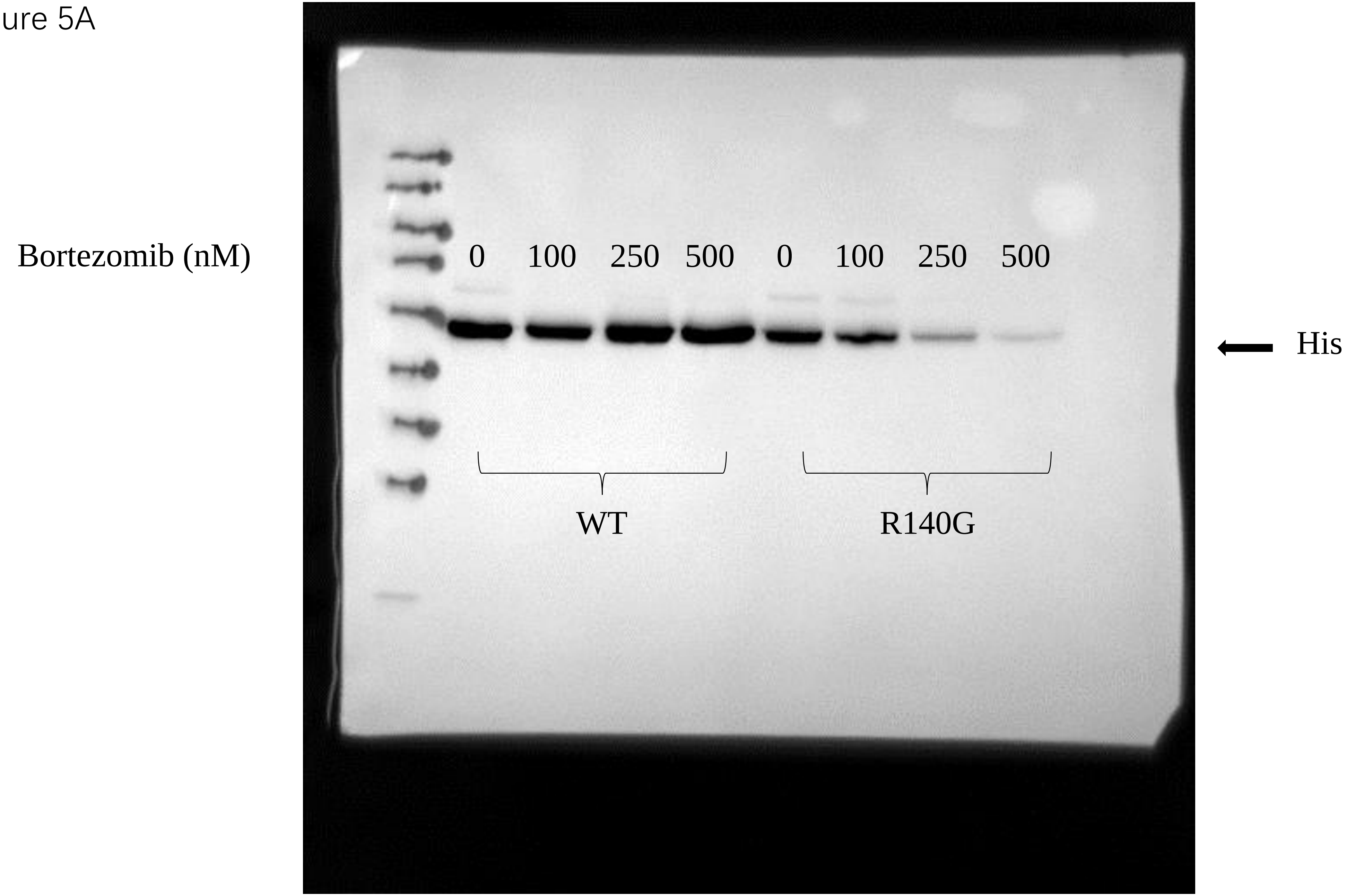

Figure 5A

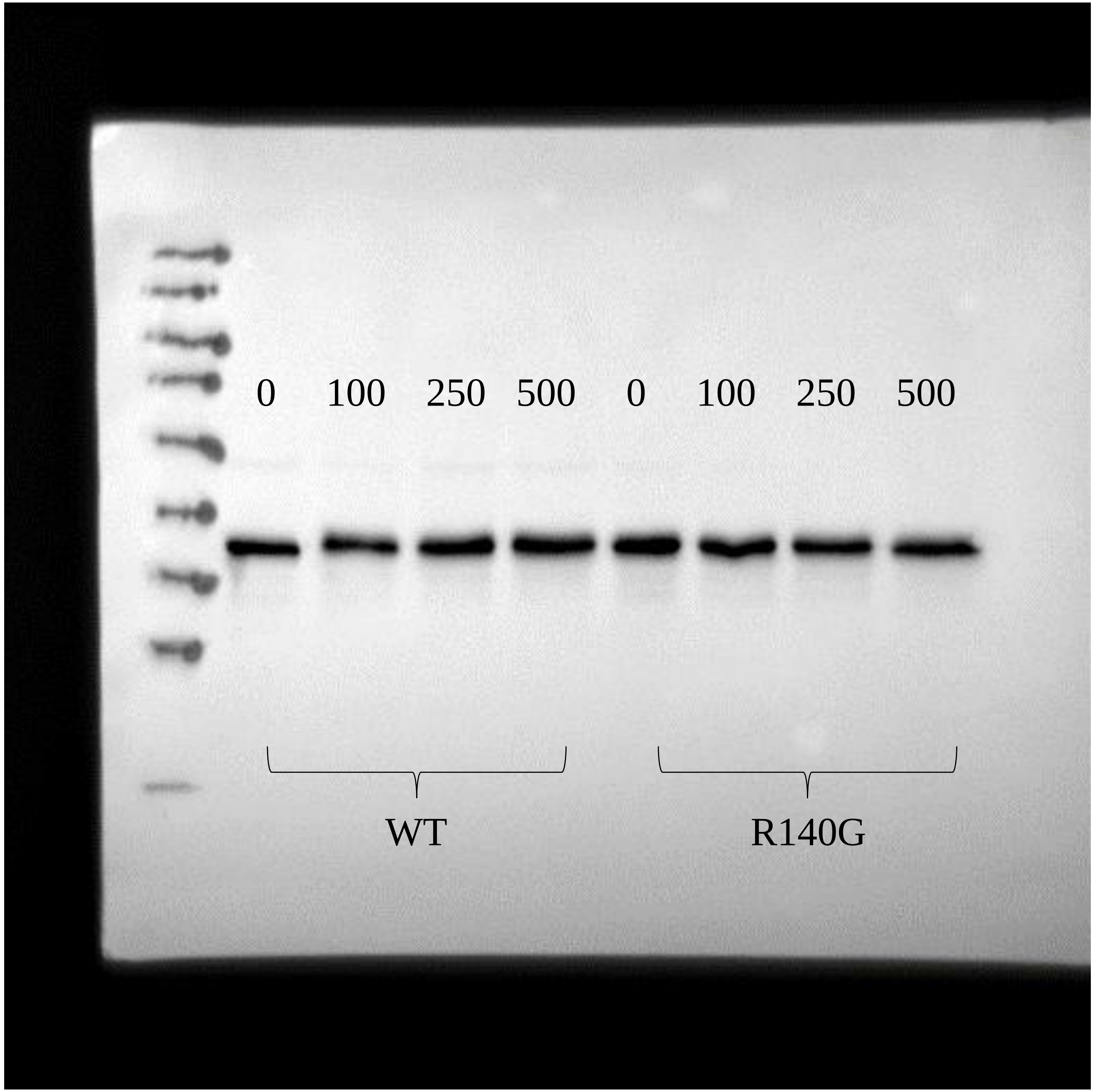

Figure 5A

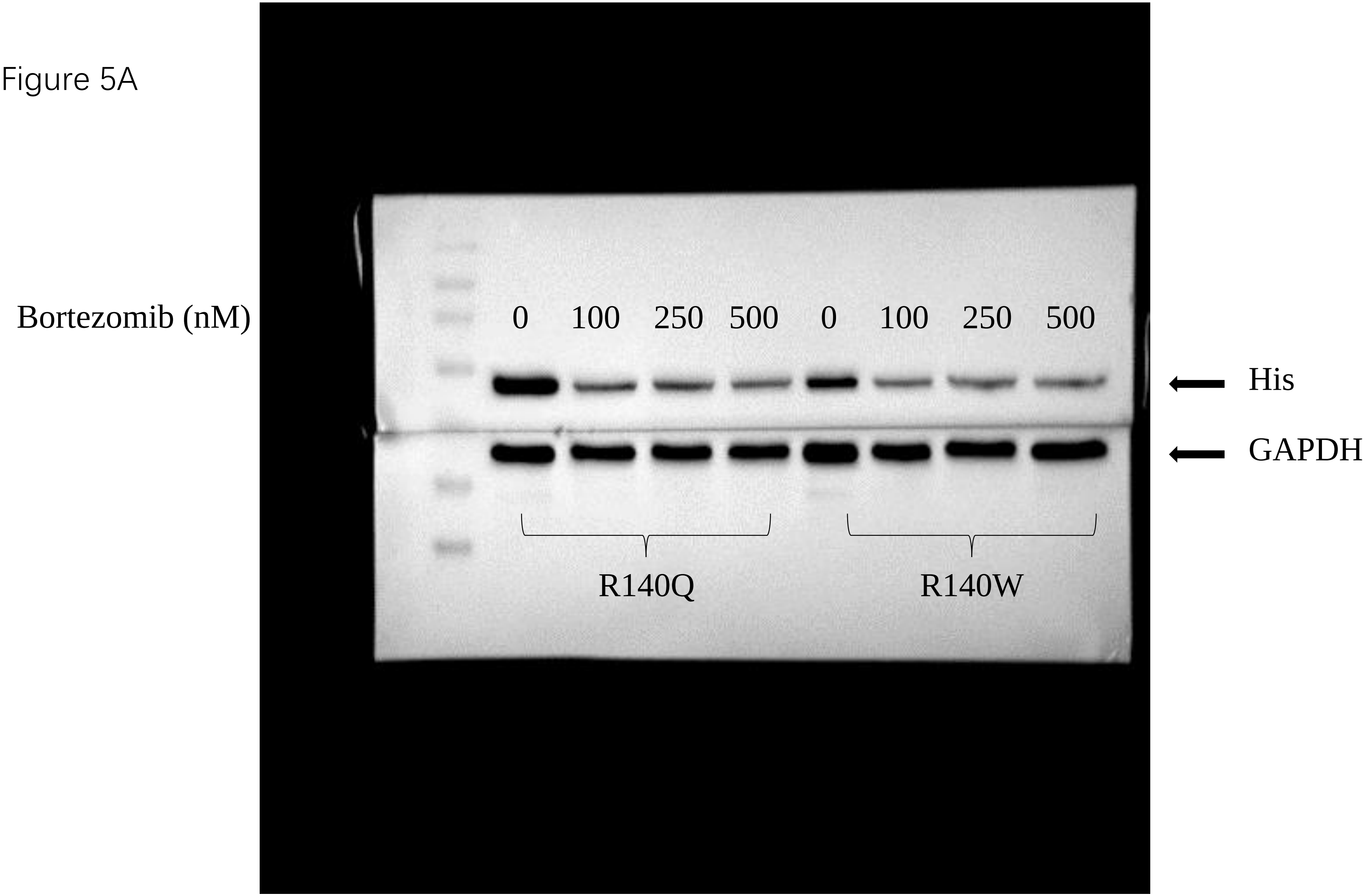

Bortezomib (nM)
0
100
250
500
0
100
250
500
His
GAPDH
R140Q
R140W

Figure 5A

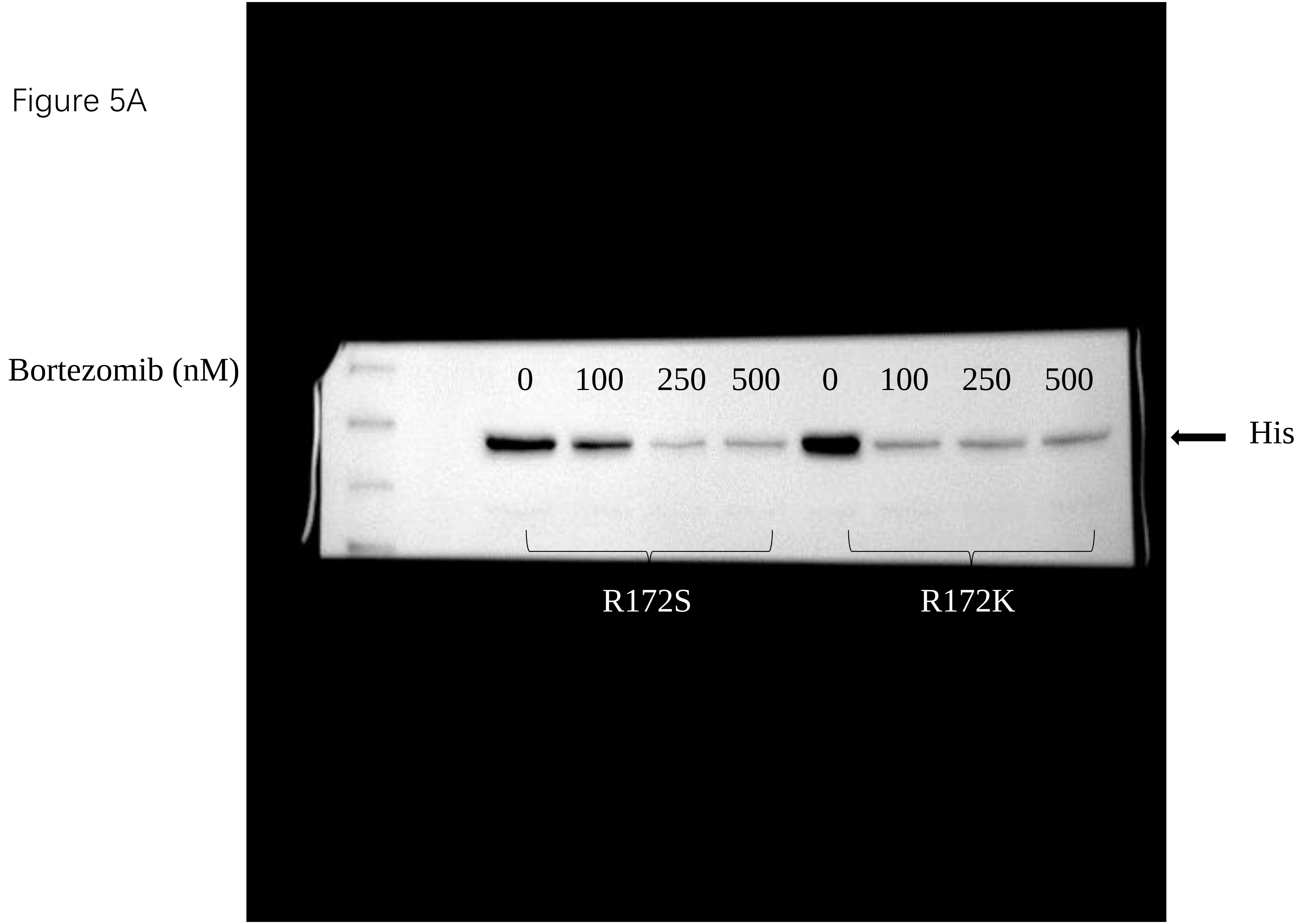

Figure 5A

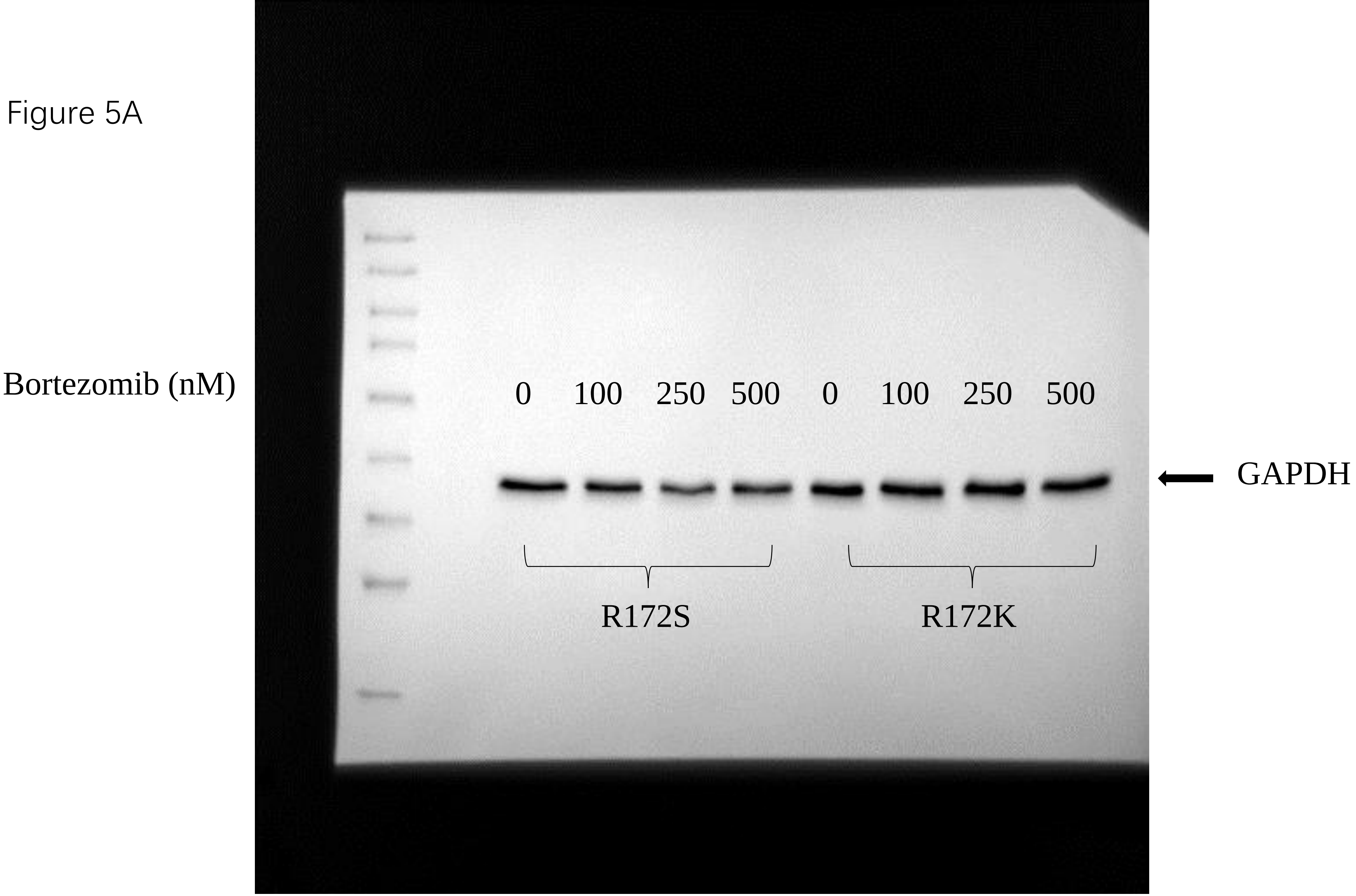

Figure 5A

Bortezomib (nM)

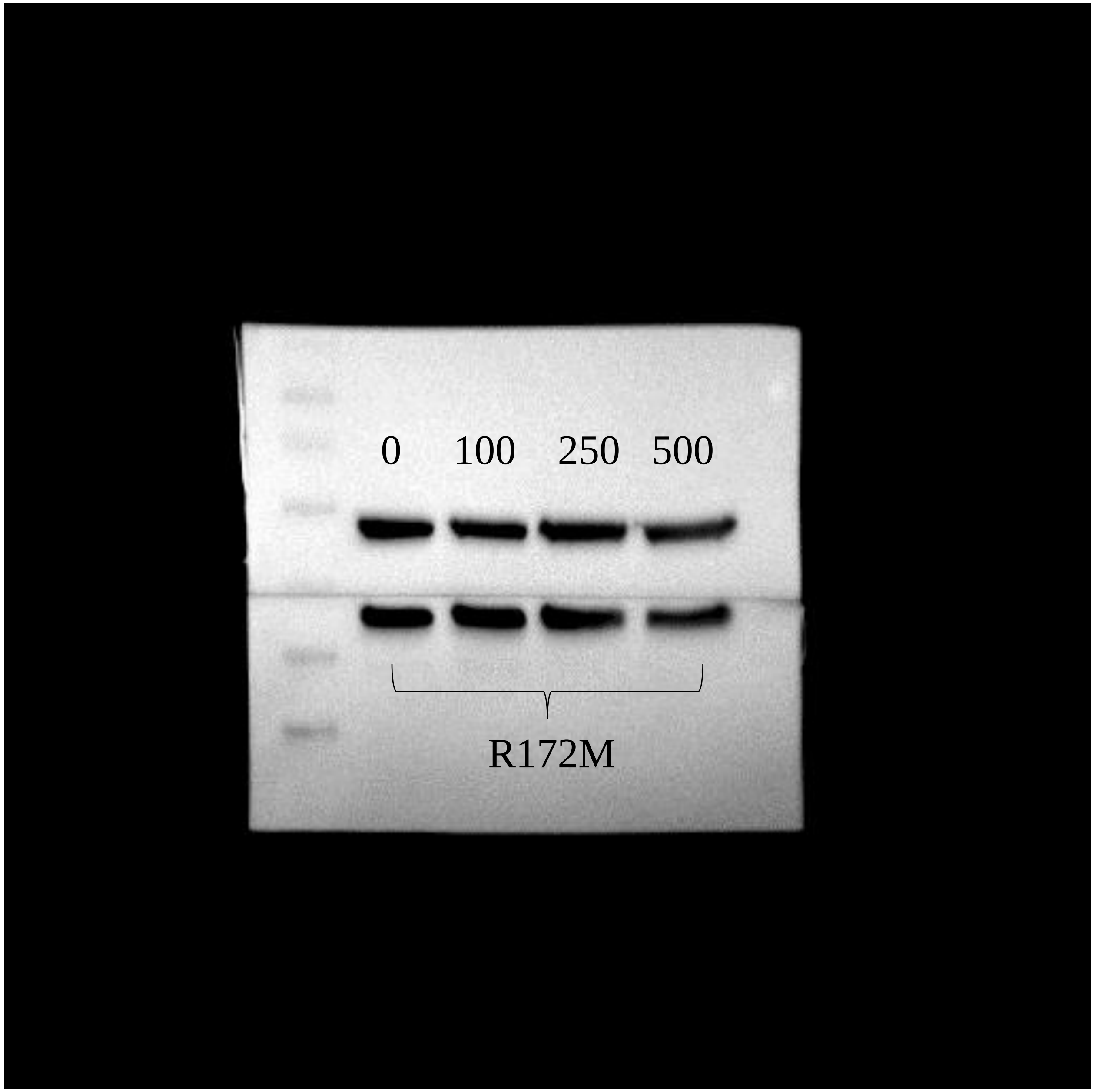

His

GAPDH

Figure 5A

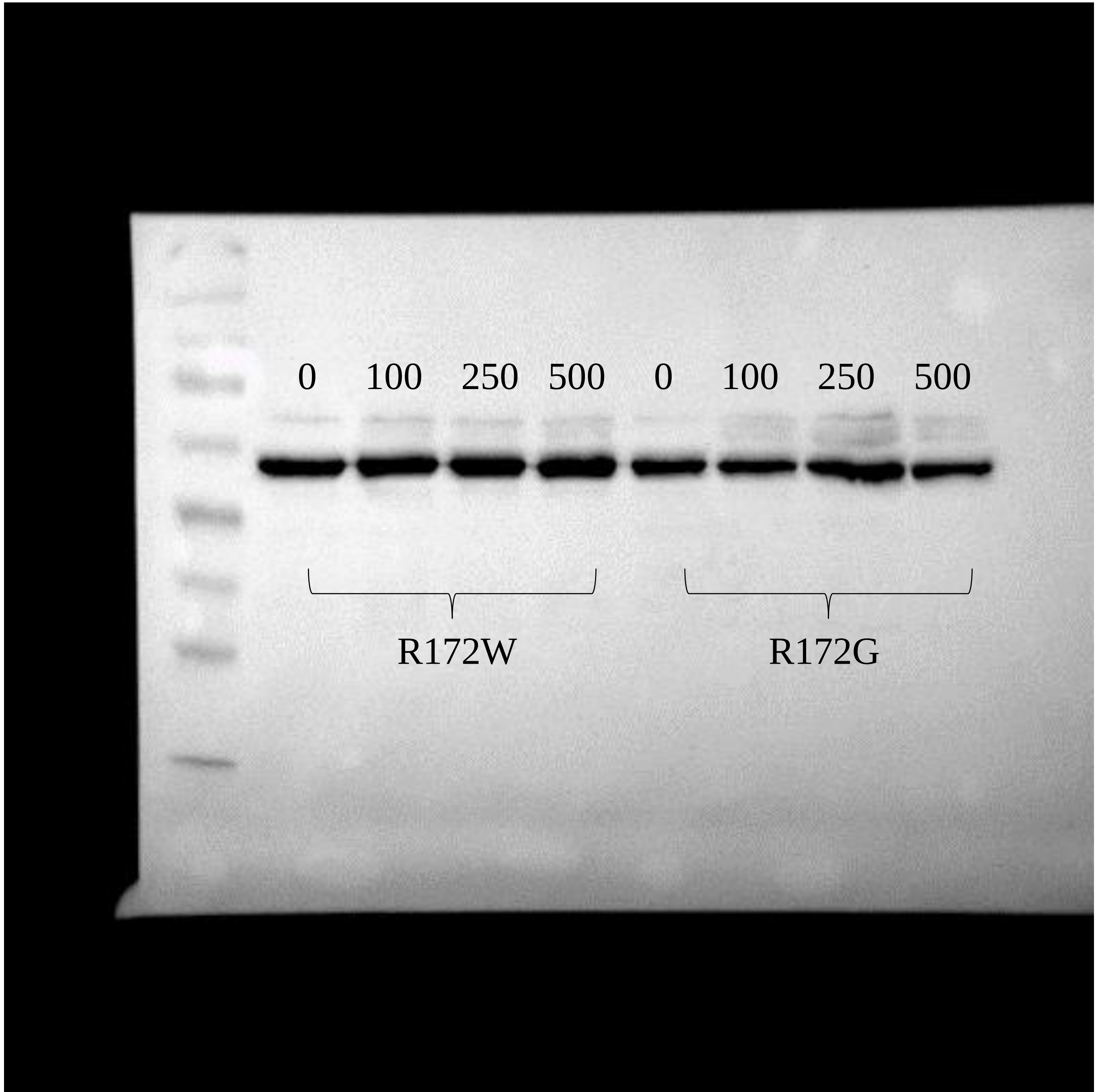

Figure 5A

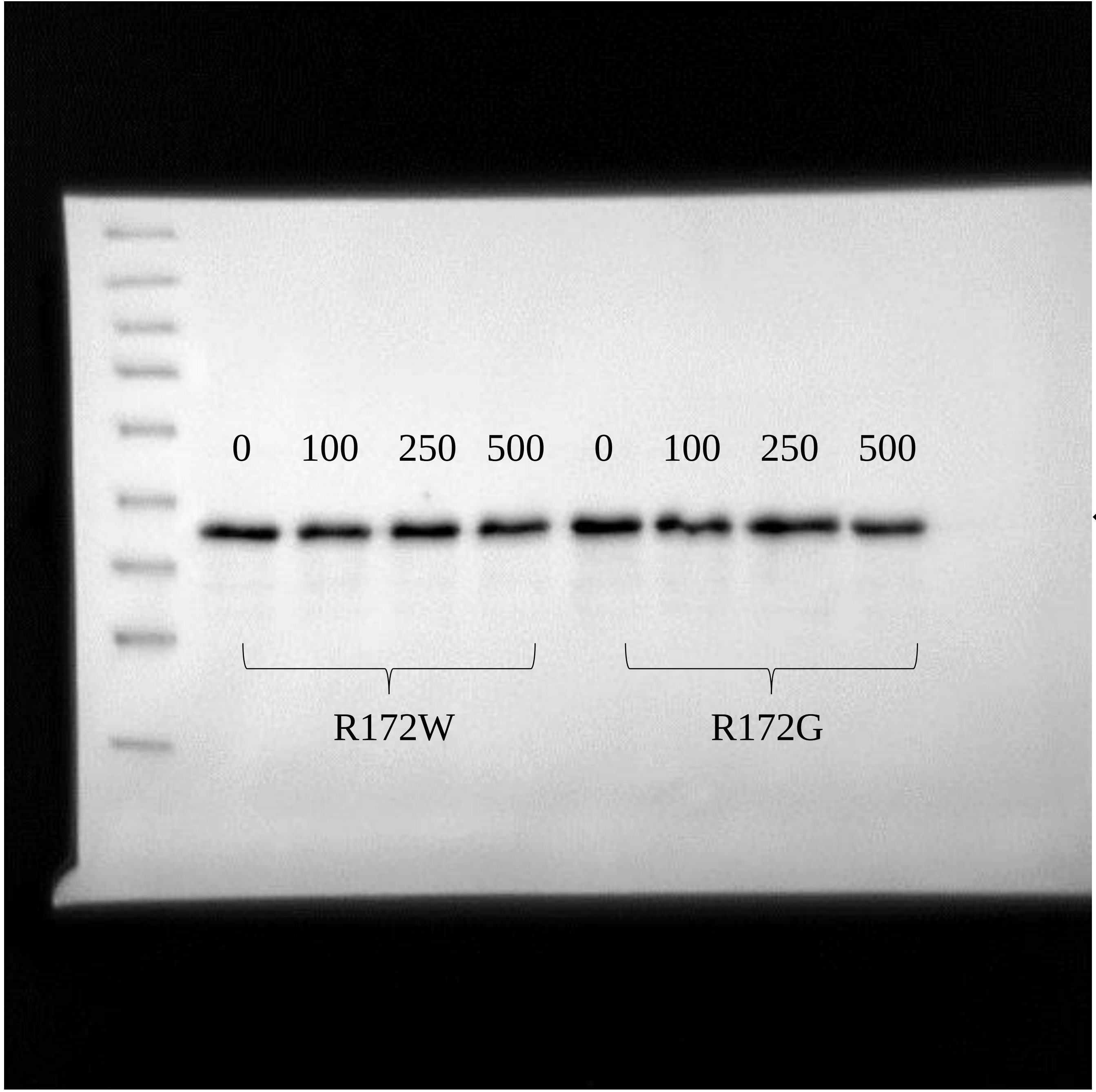

Figure 5A

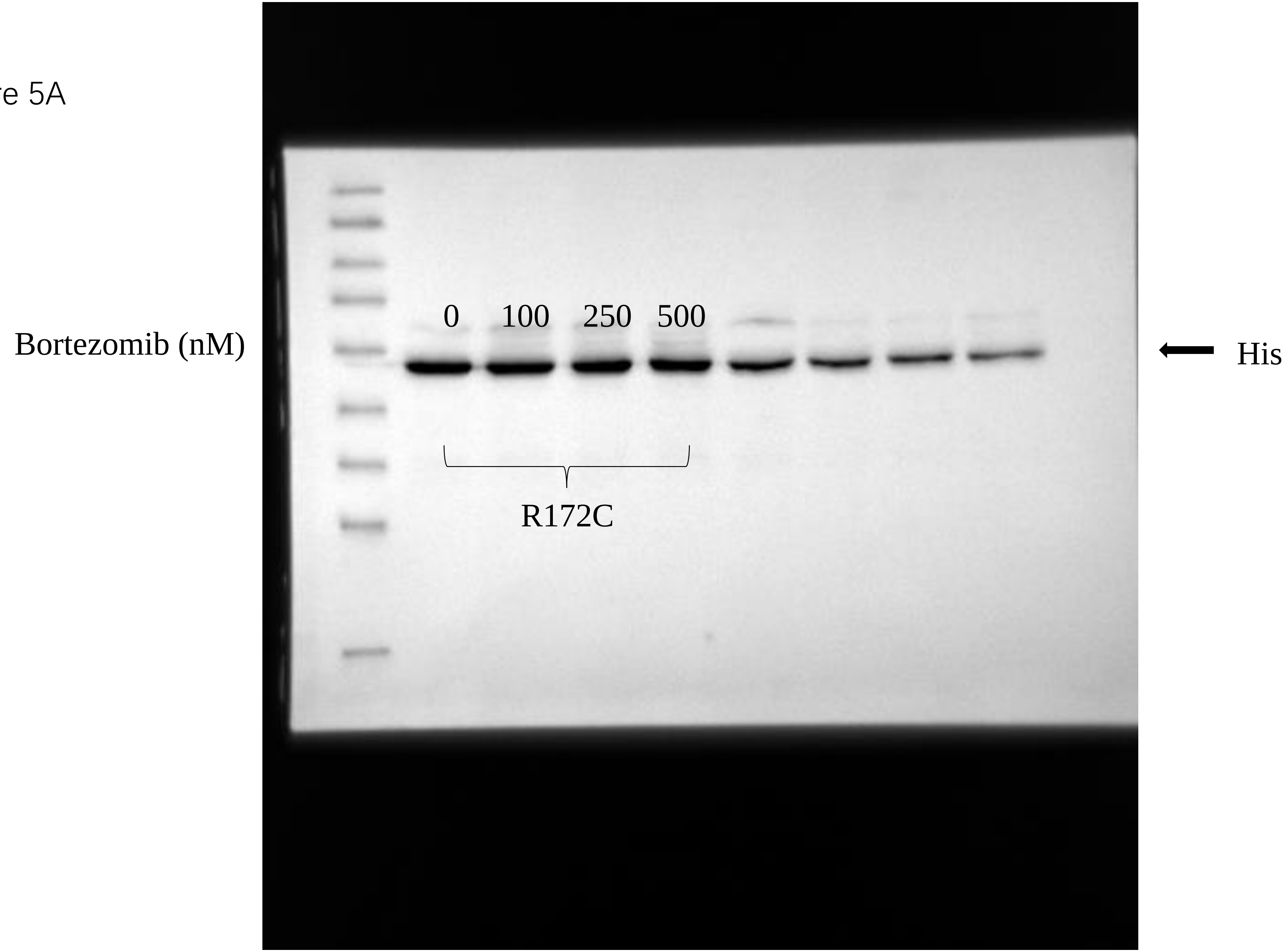

Figure 5A

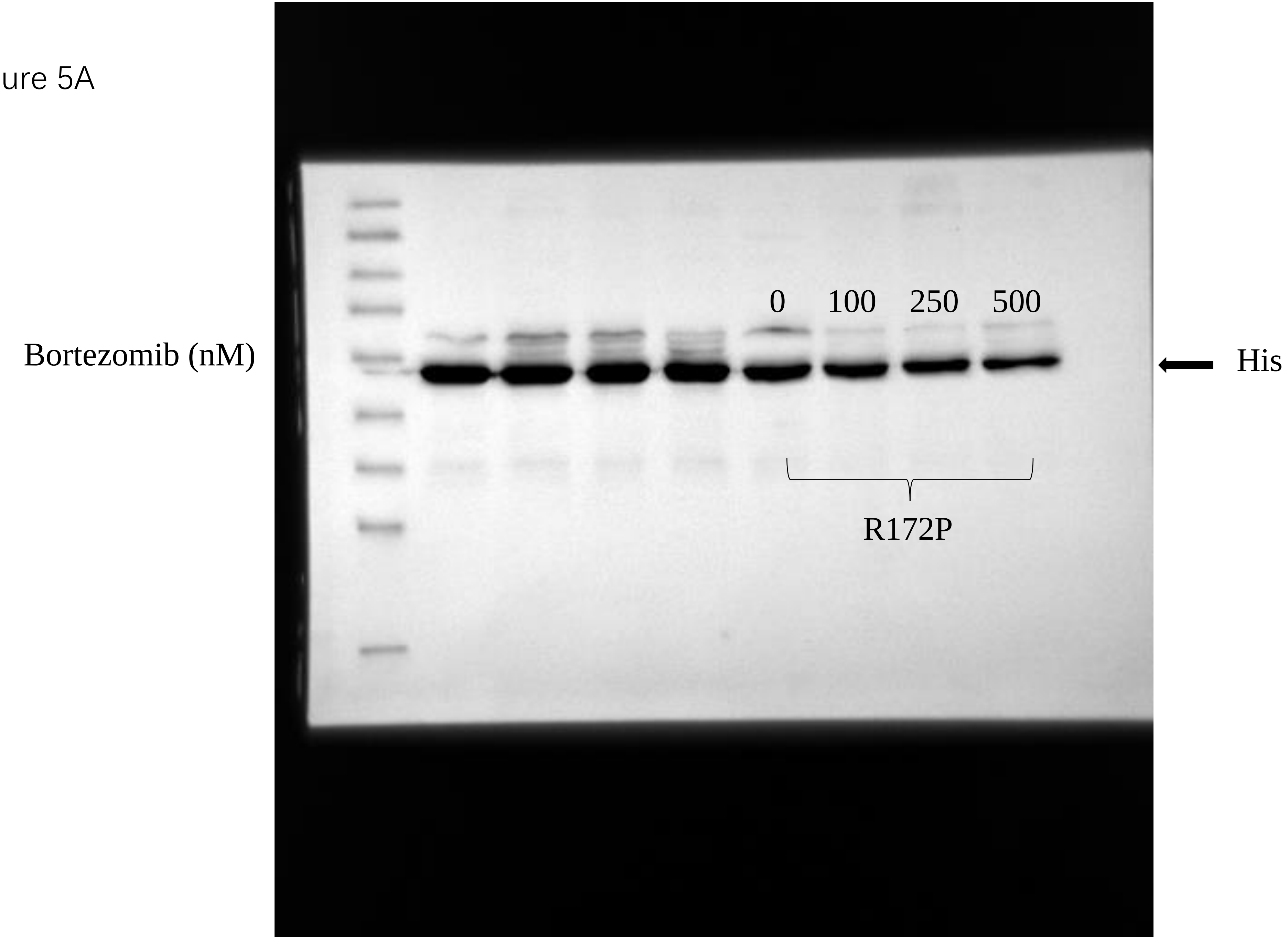

Figure 5A

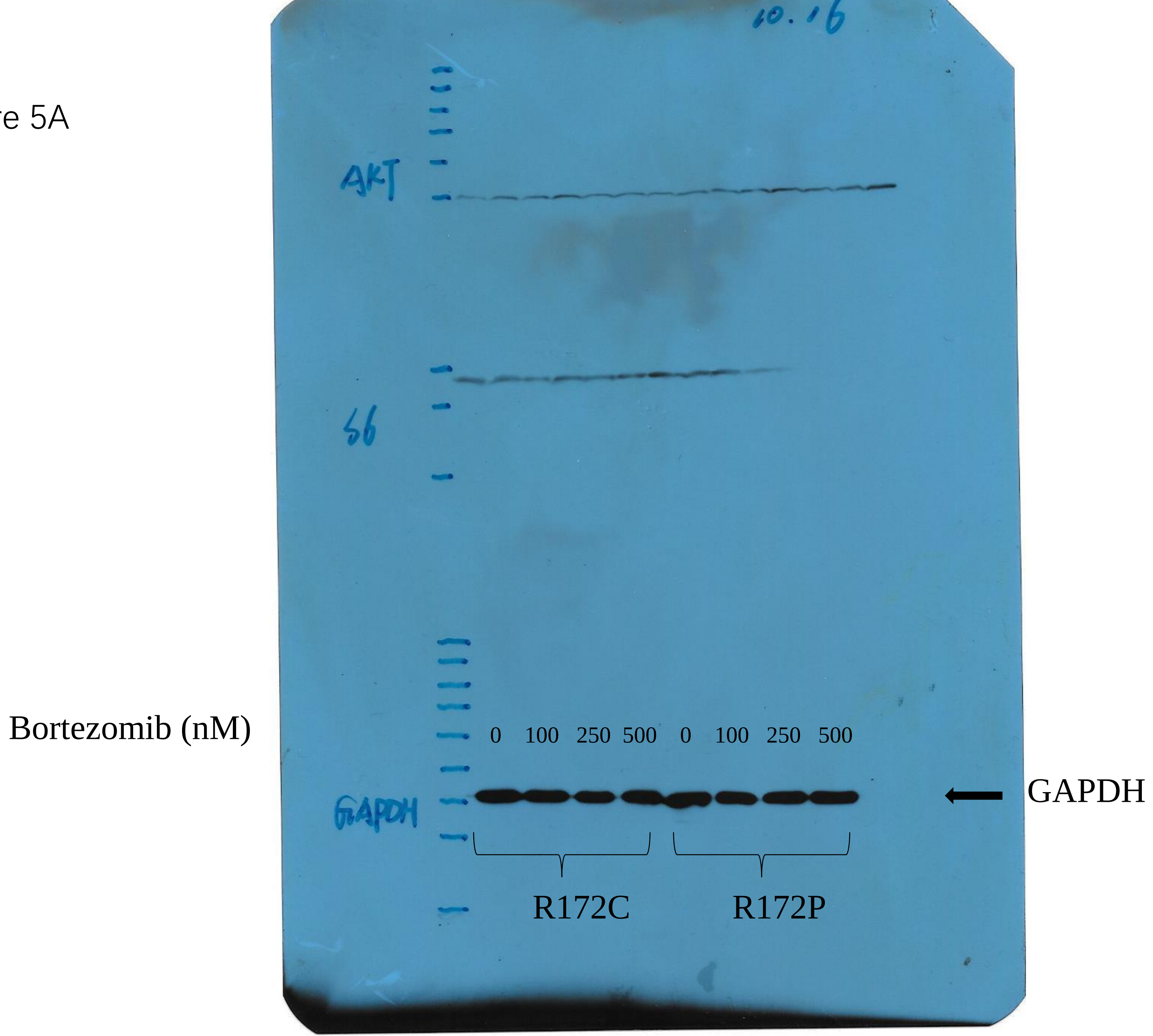
10.16
AKT
56
Bortezomib (nM)
0 100 250 500 0 100 250 500
GAPDH
GAPDH
R172C
R172P

Figure 5B

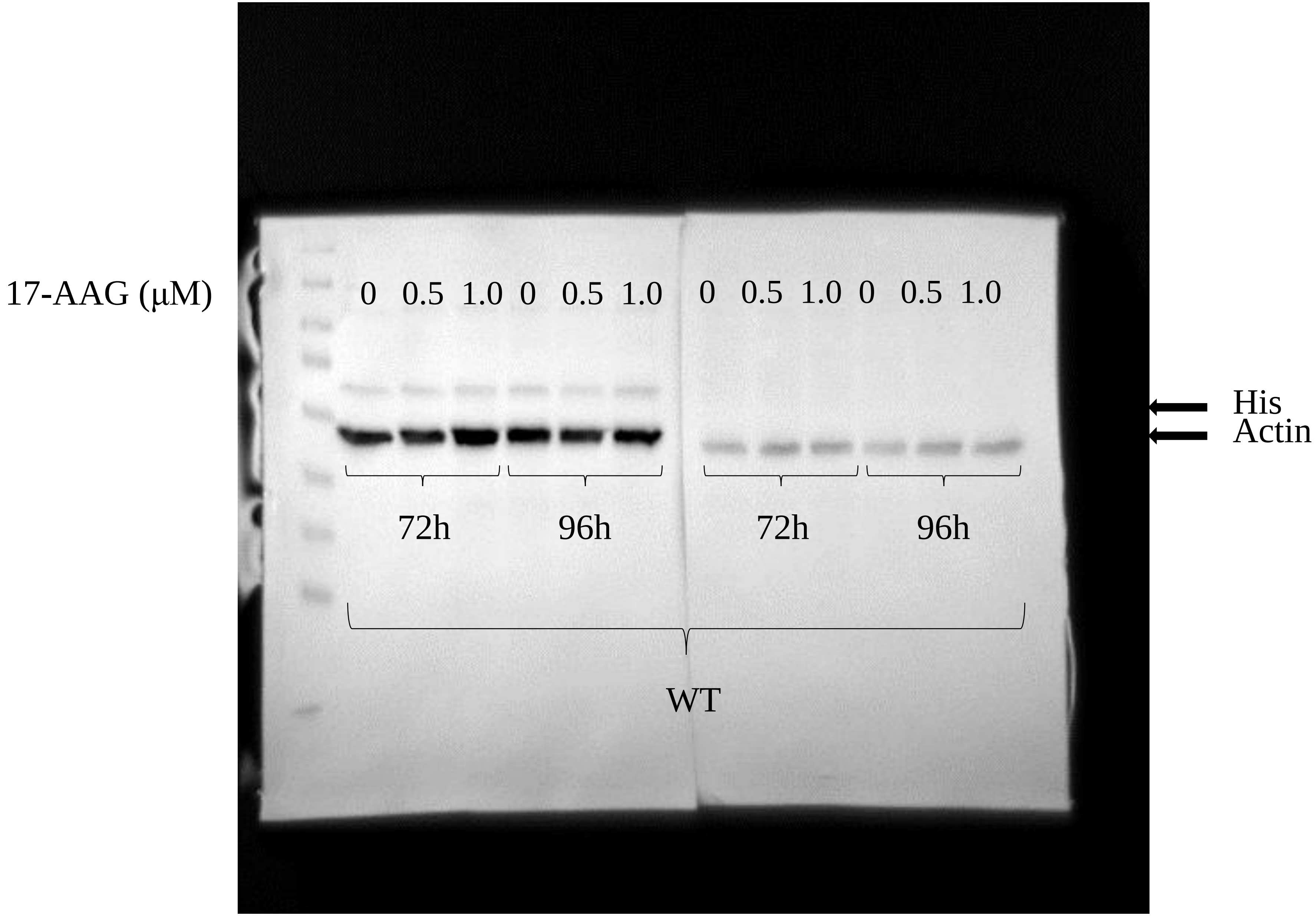

Figure 5B

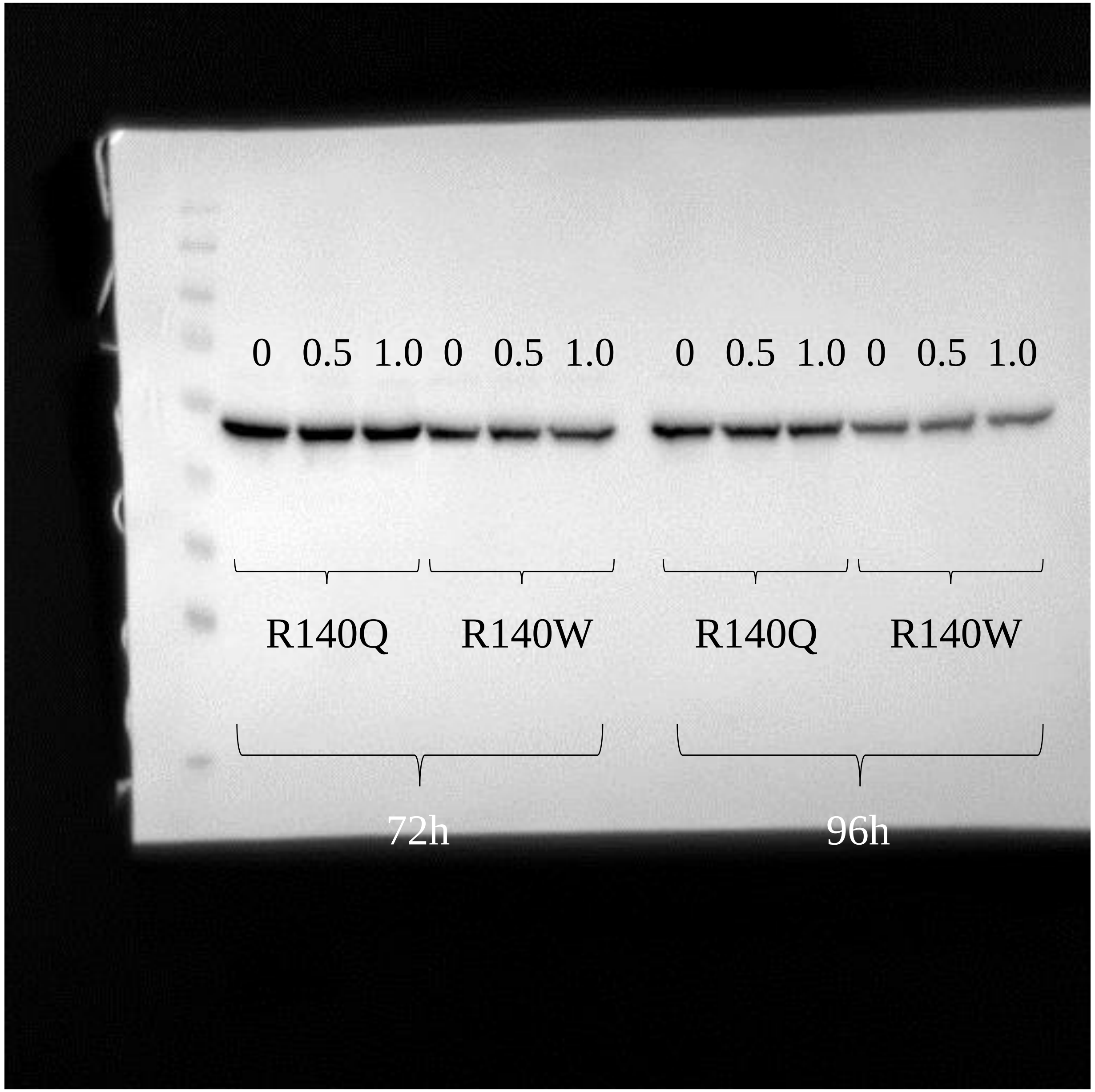

17-AAG (μM)
0 0.5 1.0 0 0.5 1.0 0 0.5 1.0 0 0.5 1.0
His
R140Q R140W R140Q R140W
72h 96h

Figure 5B

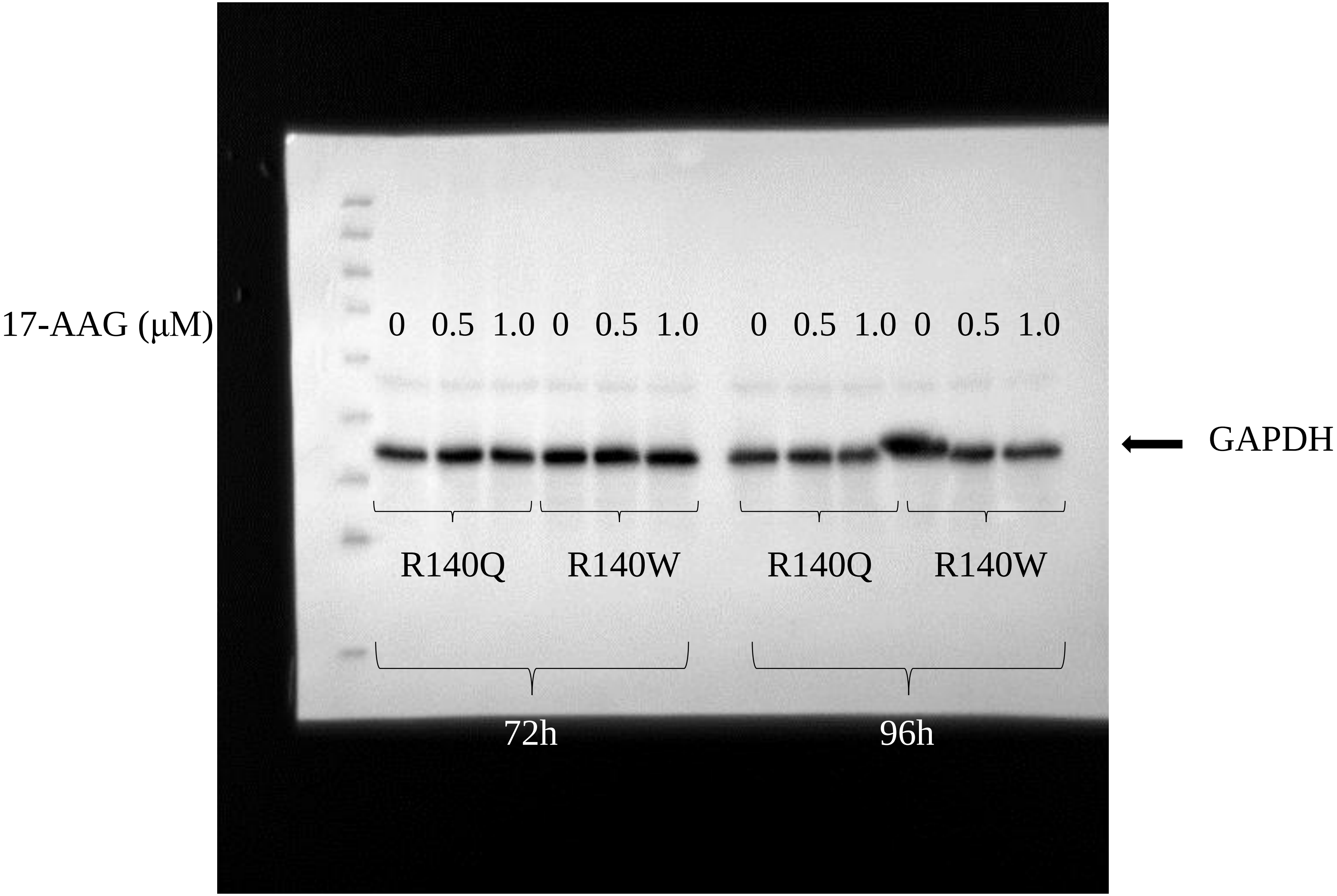
17-AAG (μM)
0 0.5 1.0 0 0.5 1.0 0 0.5 1.0 0 0.5 1.0
GAPDH
R140Q R140W R140Q R140W
72h
96h

Figure 5B

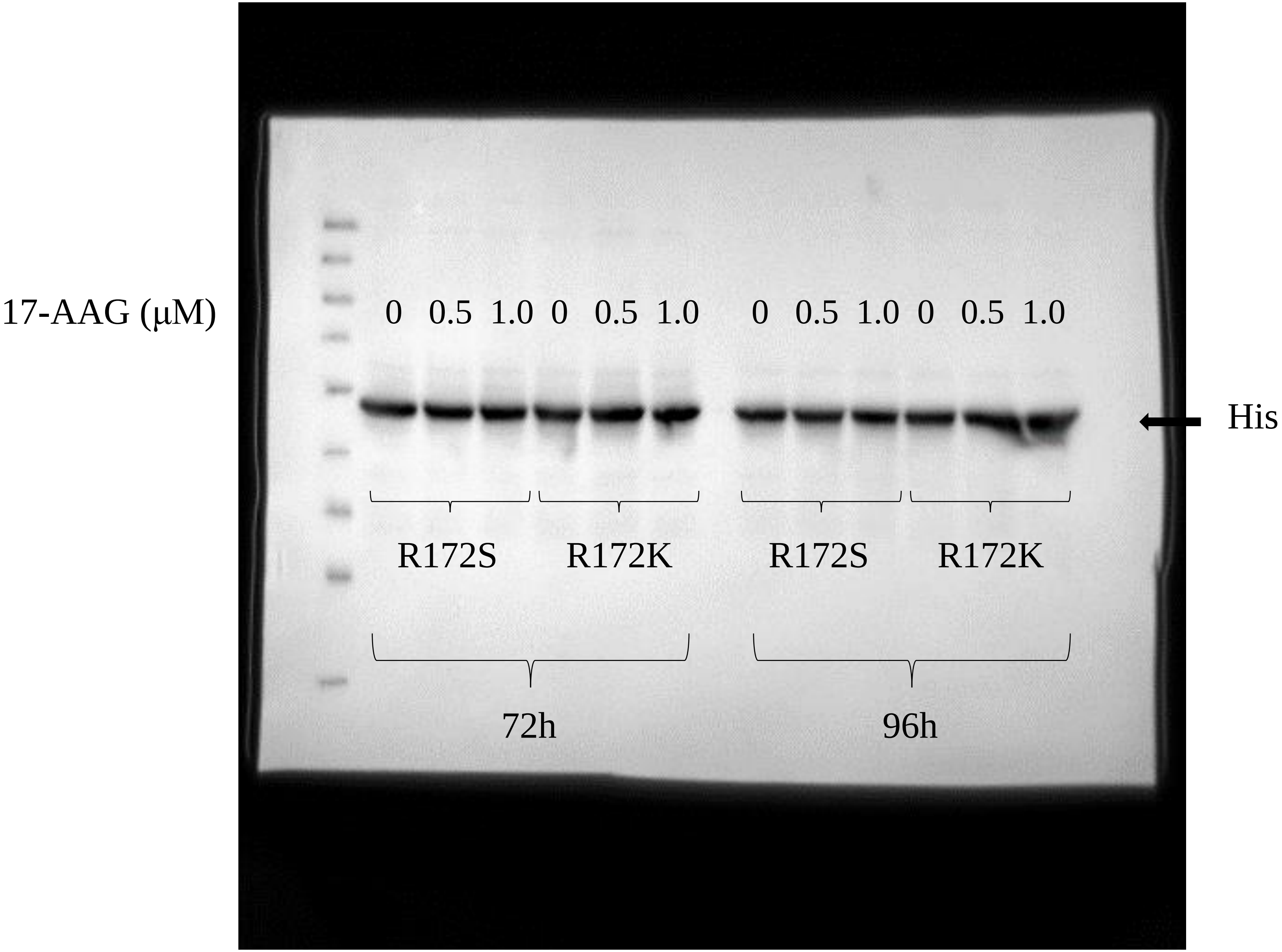
17-AAG (μM)
0 0.5 1.0 0 0.5 1.0 0 0.5 1.0 0 0.5 1.0
His
R172S R172K R172S R172K
72h 96h

Figure 5B

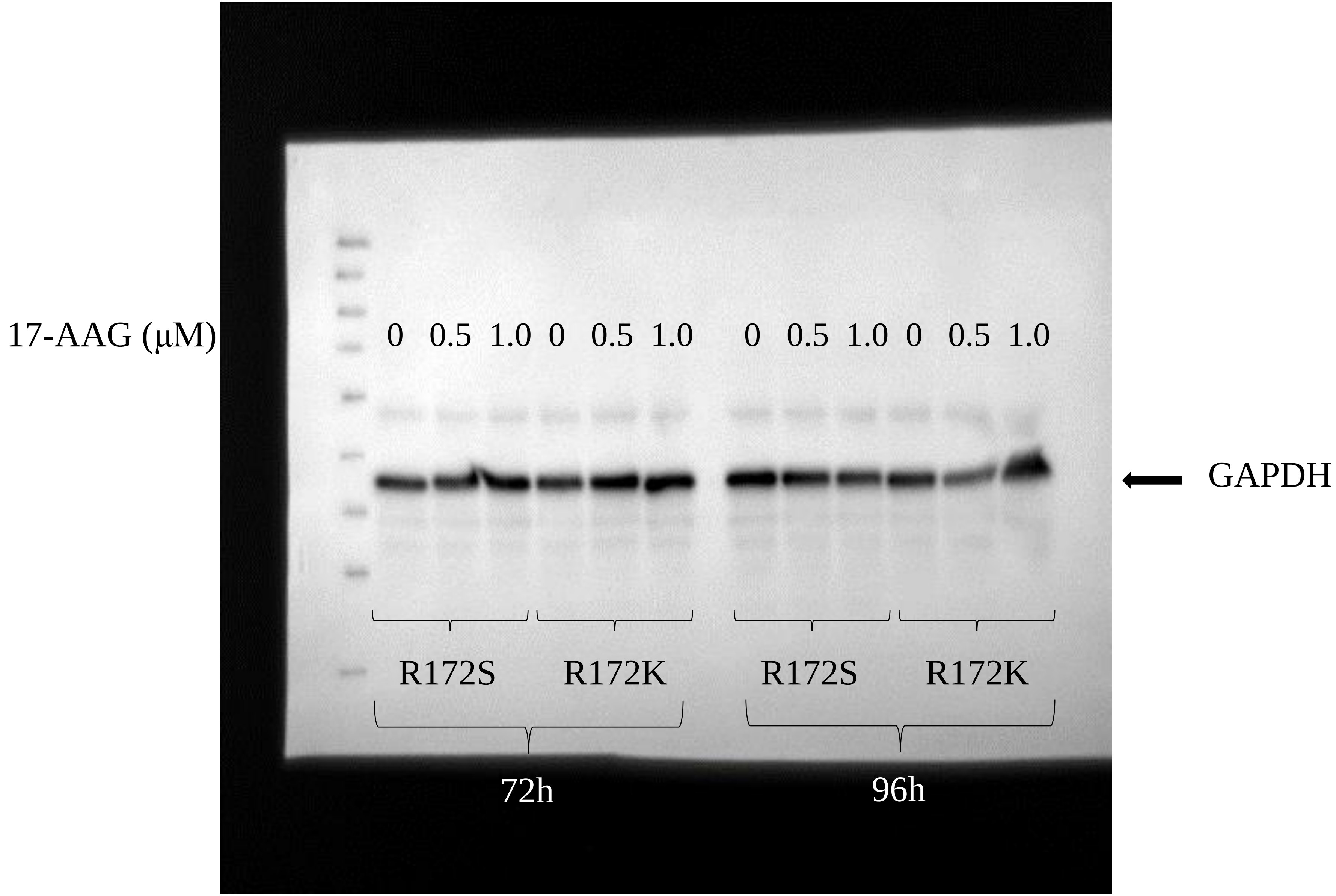
17-AAG (μM)
0 0.5 1.0 0 0.5 1.0 0 0.5 1.0 0 0.5 1.0
GAPDH
R172S R172K R172S R172K
72h
96h

Figure 5C

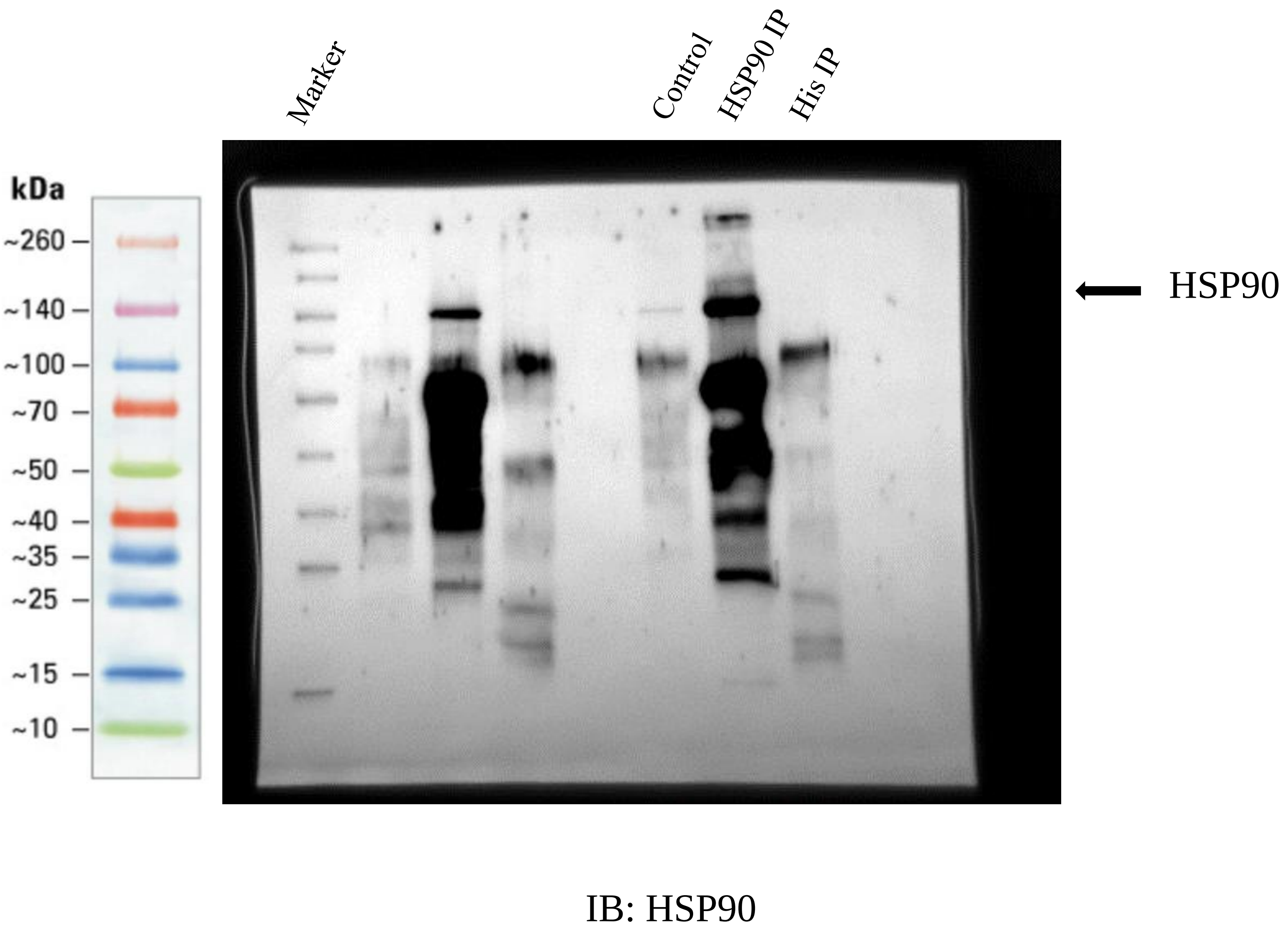
Marker
Control
HSP90 IP
His IP
kDa
~260
~140
~100
~70
~50
~40
~35
~25
~15
~10
HSP90
IB: HSP90

Figure 5C

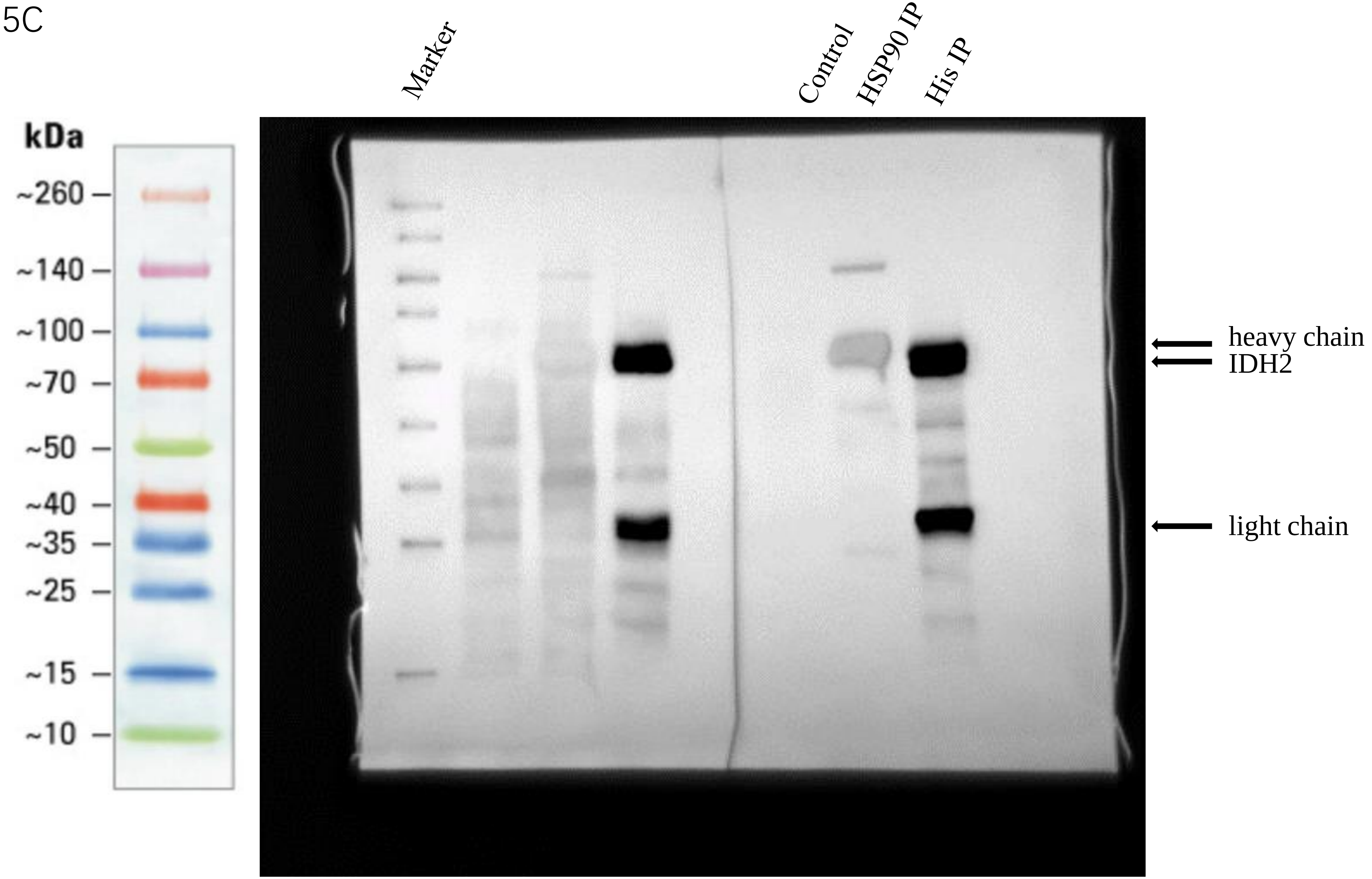

IB: His
